# Macrophage-mediated tumor homing of hyaluronic acid nanogels loaded with polypyrrole and anticancer drug for targeted combinational photothermo-chemotherapy

**DOI:** 10.7150/thno.60427

**Published:** 2021-05-13

**Authors:** Tingting Xiao, Wei Hu, Yu Fan, Mingwu Shen, Xiangyang Shi

**Affiliations:** State Key Laboratory for Modification of Chemical Fibers and Polymer Materials, Shanghai Engineering Research Center of Nano-Biomaterials and Regenerative Medicine, College of Chemistry, Chemical Engineering and Biotechnology, Donghua University, Shanghai 201620, People's Republic of China.

**Keywords:** macrophages, hyaluronic acid nanogels, polypyrrole, tumor homing property, photothermo-chemotherapy

## Abstract

**Rationale:** Development of nanosystems that can be integrated with macrophages (MAs), an emerging carrier system, for effective tumor therapy remains to be challenging. We report here the development of MAs specifically loaded with hyaluronic acid (HA) nanogels (NGs) encapsulated with a photothermal agent of polypyrrole (PPy) and anticancer drug doxorubicin (DOX) (HA/DOX@PPy NGs) for tumor homing and combination photothermo-chemotherapy.

**Methods:** Cystamine dihydrochloride-crosslinked HA NGs were first prepared through a double emulsification method, then loaded with PPy via an* in-situ* oxidization polymerization and physically encapsulated with DOX. The created HA/DOX@PPy NGs were well characterized and subjected to be endocytosed by MAs (MAs-NGs). The MAs-mediated tumor-homing property, phenotype changes and photothermal performance of MAs-NGs were investigated* in vitro*, and a subcutaneous tumor model was also established to confirm their targeting capability and enhanced antitumor therapy effect *in vivo*.

**Results:** The generated hybrid NGs possess a size around 77 nm and good colloidal stability, and can be specifically endocytosed by MAs without appreciably affecting their normal biofunctionalities. In particular, NG-loaded MAs display excellent *in-vitro* cancer cell and *in-vivo* tumor homing property. Systemic administration of the MAs-NGs leads to the significant inhibition of a subcutaneous tumor model through combination photothermo-chemotherapy under laser irradiation.

**Conclusions:** The developed hybrid HA-based NG nanosystem incorporated with PPy and DOX fully integrates the coordination and heating property of PPy to regulate the optimized DOX release in the tumor region with the assistance of MA-mediated tumor homing, providing a promising cell therapy strategy for enhanced antitumor therapy.

## Introduction

Owing to the rapid development of nanotechnology in medical applications, nanoparticle (NP)-based drug delivery systems have shown a great potential for selective transportation of therapeutic or diagnostic agents to tumors 1. In general, nanomaterials can be accumulated in solid tumors through the passive targeting strategy based upon enhanced permeability and retention (EPR) effect of tumor vasculature and lymphatic drainage 2, 3. Numerous efforts and substantial advancements have been made in designing a wide variety of NP systems to improve their transportation efficiency toward tumor tissues 4-6. On the other hand, active targeting based on the high affinity of targeting ligands linked to NPs to specific receptors on the cancer cell surface become another common strategy 7. However, the synthetic organic, inorganic, or organic/inorganic hybrid nanocarriers generally possess “non-self” properties, and are prone to be captured by the reticuloendothelial system (RES) organs. Moreover, the obstacles such as body's own physiological barriers, tumor heterogeneity, abnormal tumor microenvironments including hypoxia, insufficient blood supply and increased interstitial fluid pressure, have seriously hindered the efficient tumor penetration of the NPs, thereby limiting their antitumor efficacy 8.

In this regard, endogenous natural cells or cell membranes have been recognized as “self” by the body due to the similar membrane structure to somatic cells. Hence, NPs loaded within cells or coated with cell membranes are able to escape from the biological barriers, thus decreasing the possible immunogenicity and toxic side effects for effective tumor delivery and therapy 9-12. Notably as reported in the literature 13, compared with the cell membrane-coated NPs, the live cells loaded with NPs exhibit a higher accumulation in the target site, which is likely due to the more active recruitment of live cell carriers to the inflammation sites. However, the live cells (e.g., macrophages (MAs)) are more inclined to be activated by cytokines, leading to local inflammation, whereas the membrane-coated NPs could not be activated because of the lack of cellular activity. In any case, due to the excellent tumor homing property, some types of cells have been employed to load NPs for specific delivery to tumors or metastatic tumor cells for improved imaging and therapy 14-16. For instance, utilizing the native recruitment of neutrophils (NEs) to inflammation site, Zhang and coworkers were able to effectively deliver paclitaxel (PTX)-loaded liposomes across the blood-brain barrier for brain tumor treatment to suppress the recurrence of glioma after surgical resection 17. In a latter study, the same group also exploited the NEs loaded with a liposomal formulation of PTX to migrate to the inflamed tumor area produced by photothermal therapy (PTT) and to significantly enhance the antitumor efficacy with reduced systemic toxicity 16.

Alternatively, as a type of natural phagocytes, MAs also have a great potential in cancer drug delivery owing to their intrinsic phagocytotic capability as well as tumor-homing property 18. In a recent study 19, Xie *et al.* reported that MAs are able to recognize cytokines of colony-stimulating factor-1, vascular endothelial growth factor, tumor apoptotic factor and interleukin secreted by tumor cells and home to tumor site for MAs-mediated delivery of silica-based drug nanocapsules to effectively kill tumors. Notably, the currently developed MA-based delivery systems are mainly focused on the single drug-loaded NPs with limited loading of therapeutics within the MAs due to the inherent toxic effect of the drugs, and it has been difficult to avoid the toxic effects to the live cell carriers caused by uncontrollable drug leakage during transportation.

In order to achieve improved therapeutic efficacy, it is essential to develop a cell-mediated multitherapy nanoplatform carrying with different therapeutic components without affecting the normal functions of MAs. Among the many different nanoplatforms, nanogels (NGs) represent a versatile platform that can be encapsulated with hydrophilic or hydrophobic therapeutics within their crosslinked networks for biomedical applications. This is mainly owing to their excellent biocompatibility, good water solubility, stimuli-responsive behavior and good fluidity, allowing for efficient cellular uptake and tumor penetration 20-22. For instance, our previous work has shown that poly(N-vinylcaprolactam) NGs with redox-responsiveness can be developed to incorporate manganese dioxide NPs and anticancer drug doxorubicin (DOX) for MR imaging-guided and ultrasound-enhanced tumor chemotherapy 23. In another work 24, our group has developed γ‑polyglutamic acid NGs loaded with polypyrrole (PPy) that can be used for radiotherapy-sensitized tumor PTT. However, these studies have not been involved in the employment of MAs to take their advantages of tumor homing property.

Furthermore, for efficient MA uptake of NGs, it would be ideal to select NGs with specific components that can target to MAs. Hyaluronic acid (HA), as a biocompatible and biodegradable polysaccharide, has been demonstrated to be efficiently internalized by MAs due to its high affinity interaction with CD44 receptor overexpressed on the MA membranes 25, 26. Hence, it is logical to hypothesize that HA NGs loaded with multiple therapeutic components and limited drug leakage during transportation could be developed to be efficiently loaded within MAs through a receptor-mediated manner for further targeted anticancer drug delivery.

**Scheme 1:**
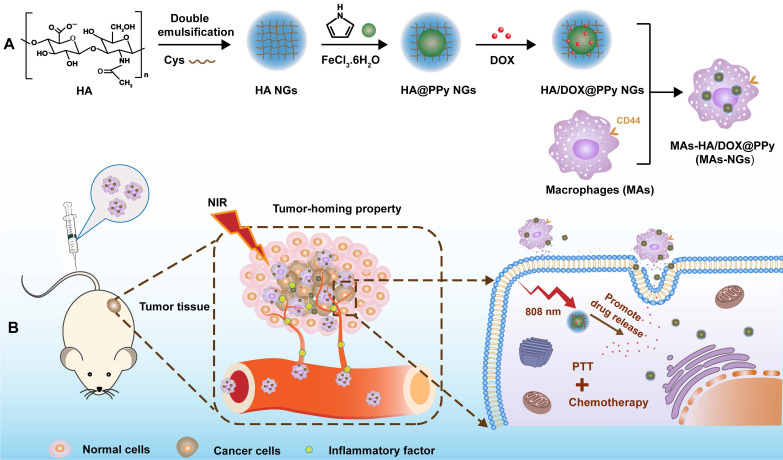
Schematic design of MAs internalized with HA/DOX@PPy NGs (MAs-NGs) for MA-mediated tumor delivery and combination PTT/chemotherapy of tumors.

Here, we report a unique design of an updated hybrid NG system for effective MA-mediated tumor delivery and enhanced tumor therapy. First, cystamine dihydrochloride (Cys)-crosslinked HA NGs were prepared using a double emulsification method, loaded with PPy NPs through *in-situ* oxidation polymerization of pyrrole (Py) monomers, and physically encapsulated with DOX through π-π stacking with the hydrophobic PPy NPs 27. After that, the developed HA/DOX@PPy NGs were loaded within MAs under regular cell culture conditions, and the formed NG-loaded MAs (MAs-NGs) were used to treat a subcutaneous tumor model for targeted combinational PTT/chemotherapy of tumors (Scheme 1). The major innovation of the current study lies in the following aspects: 1) the utilization of HA NGs can improve their loading within MAs through a receptor-mediated manner; 2) the use of PPy NPs to coordinate DOX can effectively enhance the loading of DOX within the NGs, avoid the leakage of DOX during the transportation of MAs-NGs to relieve the toxic effect to MAs, and enable combination PTT/chemotherapy of tumors by virtue of their excellent photothermal conversion efficiency under laser irradiation; and 3) under the PTT condition, DOX release can be accelerated to achieve improved tumor therapeutic efficacy.

## Results and Discussion

### Preparation and characterizations of HA/DOX@PPy NGs

**Figure 1:**
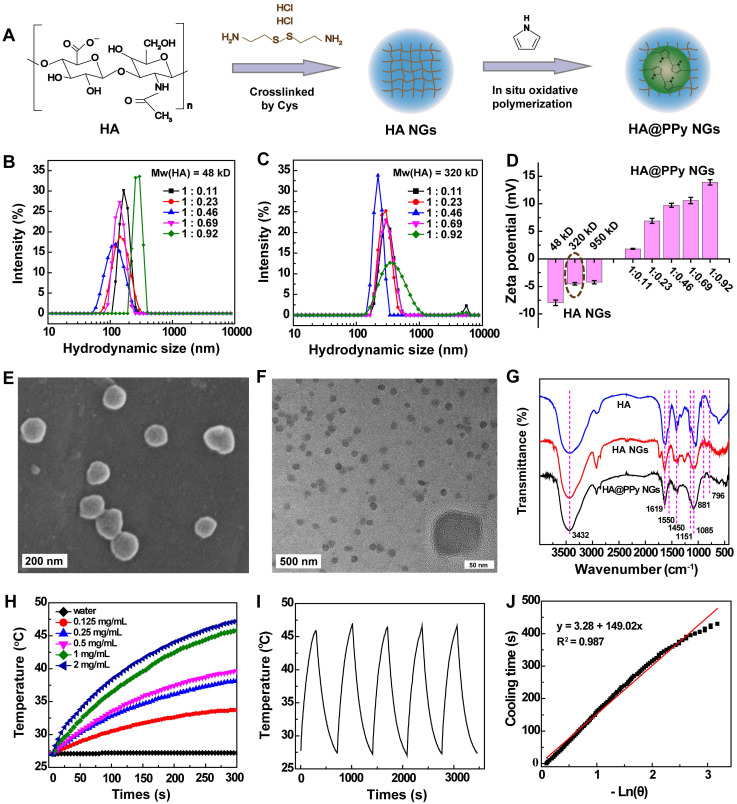
Synthesis process of HA@PPy NGs (A). The hydrodynamic size of HA@PPy NGs prepared with HA Mw at 48 kD (B) and 320 kD (C) with different PPy loading degrees. Zeta potential variations of HA NGs prepared with different Mws of HA and HA@PPy NGs with different PPy loading degrees prepared using HA with an Mw of 320 kD (D). SEM image (E) and TEM image (F) of HA@PPy NGs prepared by the 320-kD HA at the HA/Py mass ratio of 1: 0.46. FTIR spectra of HA, HA NGs and HA@PPy NGs (G). Temperature changes of HA@PPy NGs in water solution at varying concentrations (0, 0.125, 0.25, 0.5, 1 or 2 mg/mL) after an 808-nm laser irradiation for 300 s at a power density of 1 W/cm^2^ (H). Photothermal stability of the NGs after 5 cycles of laser-on and laser-off process (I) and plot of the cooling time *vs* -ln (θ) on the basis of linear regression analysis (J).

The synthesis procedure of HA/DOX@PPy NGs is shown in Scheme 1. Crosslinked HA NGs were first prepared and incorporated with positively charged Py monomers for oxidization polymerization to form HA@PPy NGs according to the literature (Figure 1A) 24. To optimize HA@PPy NGs with desired morphology and stability, we first prepared HA NGs using HA with different molecular weights (Mw = 48, 320 or 950 kD). Dynamic light scattering (DLS) and zeta potential analysis were used to assess the as-prepared HA NGs. As shown in Table S1, the hydrodynamic size of HA NGs was 243.8 ± 3.45, 316.7 ± 11.66 and 383.0 ± 5.86 nm, respectively for HA Mw of 48, 320 and 950 kD, displaying an increasing trend with the increase of Mw. Zeta potentials of the formed HA NGs also show a slight increasing trend with the HA Mw. However, obvious demulsification occurred during double emulsification process for the HA Mw of 950 kD, which might be attributable to the excessively high viscosity of HA. Accordingly, HA NGs prepared with Mws of 48 and 320 kD were selected for following PPy loading.

Different Py loadings (mass ratio of HA NGs/Py = 1: 0.11, 1: 0.23, 1: 0.46, 1: 0.69 or 1: 0.92) were investigated using HA NGs prepared with Mws of 48 or 320 kD. The hybrid NGs were examined by DLS (Figure 1B-C) and zeta potential (Figure 1D) measurements. With the increase of Py feeding, both the gradually increased surface potentials (Table S2) and improved characteristic UV absorption in an NIR region (Figure S1) verified the increased loading content of PPy within HA NGs. Interestingly, it can be seen from Table S2, Figure 1B and Figure 1C that the hydrodynamic size of the as-prepared HA@PPy NGs first decreases with the increase of PPy loading (mass ratio of Py to HA ≤ 0.46), which should be ascribed to the compression of NGs by the electrostatic interaction between negatively charged HA NGs and interior positively charged PPy NPs. However, the hydrodynamic size of the NGs increases upon further increase of Py (mass ratio of Py to HA > 0.46), possibly due to the structural expansion of NGs by solid PPy NPs. Simultaneously, it was observed that slight precipitation of NGs appeared after storage for a few days, and the lyophilized NGs were hardly redispersed in water for PPy-loaded HA NGs (Mw of HA = 48 kD) at Py/HA mass ratio of 0.46 or above. In contrast, HA@PPy NGs (Py/HA = 0.46) synthesized by 320-kD HA showed an excellent stability and redispersibility in water, PBS and complete DMEM over a period of 5 days (Figure S2). Hence, with a small hydrodynamic size (198.1 ± 4.91 nm), HA@PPy NGs prepared by the 320-kD HA at the HA/Py mass ratio of 1: 0.46 were chosen for the following studies.

Various characterizations were carried out to investigate the hybrid HA@PPy NGs. As shown in Figure S1, compared with naked HA NGs, the broad NIR absorption of HA@PPy NGs can be seen, proving the successful loading of PPy within the HA NGs. Additionally, thermal gravimetric analysis (TGA) was used to determine the actual loading amount of PPy within the HA@PPy NGs (Figure S3). By subtracting the weight loss of HA@PPy NGs (81.1%) from that of HA NGs (93.8%) at a temperature of 800 °C, the loading percentage of PPy was calculated to be 12.7%.

Scanning electron microscopy (SEM) and transmission electron microscopy (TEM) were employed to characterize the morphology of the HA@PPy NGs (Figure 1E-F). Apparently, the HA@PPy NGs displayed a spherical shape with a monodisperse size distribution and mean dimeters of 77.3 ± 9.40 nm (SEM, Figure S4A) and 74.9 ± 6.84 nm (TEM, Figure S4B), respectively. The smaller size of NGs measured by TEM than that measured by SEM should be attributed to the SEM sample preparation protocols that are involved in the sputter coating of a thin gold film onto the sample surface. Meanwhile, it is worth noting that the sizes of HA@PPy NGs measured by SEM/TEM are much smaller than their hydrodynamic size (198.1 ± 4.91 nm) recorded by DLS. This might be due to the fact that SEM and TEM measure samples in a dried state and hence the size of shrunken NGs, while DLS measures NGs composed of a significant amount of water in aqueous solution 28, 29.

To verify the structure of HA@PPy NGs, Fourier transform infrared (FTIR) spectra of HA, HA NGs and HA@PPy NGs were recorded (Figure 1G). Compared with pure HA, the absorption bands at 1619 cm^-1^ and 1450 cm^-1^ from both HA NGs and HA@PPy NGs are respectively assigned to COO^—^ anti-symmetric (ν_as_) and symmetric stretching vibration (ν_s_), respectively, and the intensity of the peaks significantly decreases after Cys crosslinking to form the NGs 26. The bands at 1151 and 1085 cm^-1^ are associated with the stretching vibration of the primary alcohol in polysaccharide 30. Moreover, the broad and overlapping bands appearing at 3432 cm^-1^ should be attributed to the O-H symmetric stretching vibrations (ν_s_) of HA or the typical ν_s_(N-H) vibration of PPy. In addition, HA@PPy NGs exhibit some new bands after oxidative polymerization to form PPy. Among them, the absorption bands at 881 and 796 cm^-1^ are assigned to the out-of-plane vibration of =C-H in PPy rings 31, 32. These facts demonstrate the successful loading of PPy within the HA NGs.

Due to the broad NIR absorption feature of HA@PPy NGs (600-1000 nm), we next investigated their photothermal properties. The real-time temperature changes of NGs in an aqueous solution (300 μL) versus concentration were recorded after the NGs were exposed to an 808-nm laser (Figure 1H). As opposed to the control of water, the temperature of HA@PPy NGs solution increases with the laser irradiation time. The temperature change (ΔT) increases from 6.7 to 20.2 °C with the NG concentration increasing from 0.125 to 2 mg/mL, displaying a concentration dependency. At an NG concentration of 1 mg/mL, the ΔT value reaches 18.8 °C. Next, the photothermal stability of the HA@PPy NGs (1 mg/mL) was evaluated by five cycles of laser on-off processes to record their temperature changes (Figure 1I). It appears that the temperature values at each cycle of laser-on for 5 min and laser-off are quite similar, demonstrating their nice photothermal stability. Furthermore, the photothermal conversion efficiency (η) of HA@PPy NGs was calculated according to the reported literature method 33. We monitored the temperature change during a heating (laser on) and cooling (laser off) process (Figure S5), followed by linear fitting of the cooling time versus -ln(θ) to obtain the time constant τ_s_, represented with a slope of the plot in Figure 1J (149 s). Finally, η was calculated to be 52.74%, which is much higher than those of some reported inorganic photothermal agents 34, 35. Thus, HA@PPy NGs can efficiently convert NIR laser energy into heat for potential PTT applications.

HA@PPy NGs with uniform size distribution, excellent colloidal stability and photothermal conversion efficiency were next used to load anticancer drug DOX. In order to reduce the potential cytotoxicity of DOX to MAs, we selected the NG/DOX mass ratio of 1 : 0.25. UV-vis spectroscopy was used to confirm the DOX loading within the NGs (Figure S6). Compared with the DOX-free HA and HA@PPy NGs, HA/DOX@PPy NGs display a typical DOX absorption peak at 490 nm, indicating the successful loading of DOX. The drug encapsulation efficiency (EE%) and loading content (LC%) were calculated to be 95.0% and 19.2%, respectively. Notably, the HA/DOX@PPy NGs with drug loading are quite stable as demonstrated by DLS assay of their hydrodynamic sizes for a period of 5 days in different aqueous media (Figure S7), which is comparable to drug-free HA@PPy NGs. In addition, in comparison to DOX-free HA@PPy NGs with a TEM size of 74.9 nm, the drug-loaded NGs have a slightly increased size of 77.2 nm (Figure S8), further suggesting the drug loading within the NGs.

**Figure 2:**
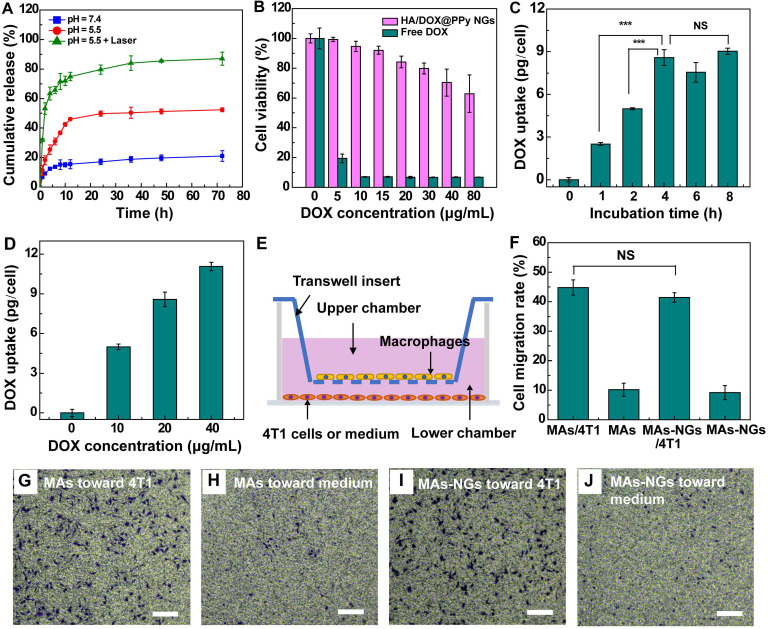
DOX release from the HA/DOX@PPy NGs at different pHs without or with an 808-nm laser irradiation for 10 min (A). CCK-8 assay of viability of MAs treated with free DOX and HA/DOX@PPy NGs for 24 h at different DOX concentrations (B). Intracellular DOX uptake by MAs after treatment with the HA/DOX@PPy NGs ([DOX] = 20 µg/mL) for different time periods (0, 1, 2, 4, 6 and 8 h, respectively) (C) or incubation with NGs at different DOX concentrations ([DOX] = 0, 10, 20 and 40 µg/mL) for 2 h (D). Schematic illustration of the transmigration assay toward 4T1 cells (E), the statistical migration percentage (F) and the micrographs of the MAs transmigrated to the lower chamber that were stained with crystal violet (G-J). Scale bar for G-J represents 100 µm for each panel. The NS means no significant difference, and ***represents p < 0.001.

The DOX release from the HA/DOX@PPy NGs under different pHs (pH 7.4 and pH 5.5) were also investigated. As reported in the literature 19, 36, it generally takes several hours for most MAs to migrate to the inflamed tumor tissues after intravenous injection. It is desired for the NP-loaded MAs to have a minimum drug release in the early hours during transportation, and to adequately release the drugs after they arrive at the tumor sites. As shown in Figure 2A, the NGs can release 13.6% DOX under pH 7.4 and 30.9% DOX under pH 5.5 at an early time point of 6 h, which is beneficial to reduce their toxic effect to the MA carriers. At 72 h, the cumulative DOX release reaches 21% under pH 7.4 and 52.4% under pH 5.5, respectively. The fast DOX release under a slightly acidic condition should be ascribed to the improved water solubility of DOX after protonation, in agreement with the literature 36, 37. However, still there is nearly half of the loaded DOX that is unable to be released under pH 5.5. Encouragingly, enhanced drug release (87%) can be achieved at pH 5.5 when the HA/DOX@PPy NGs were exposed to an 808-nm laser for 10 min. This might be due to the heat energy generated upon the NIR laser irradiation that can trigger and accelerate the release of DOX from the HA/DOX@PPy NGs 27, 36, 38. It implies that the drug release can be controlled and facilitated under an acidic tumor microenvironment with an NIR laser irradiation at the tumor sites, benefiting to reduce its toxic effect to normal tissues and to improve its therapeutic effect on tumor cells.

### *In vitro* cytotoxicity and cellular uptake assays

In order to optimize the appropriate concentrations of HA/DOX@PPy NGs to be loaded within the MAs, cytotoxicity assay of the NGs was performed after they were incubated with MAs for 24 h. Free DOX with similar concentrations was tested as a positive control (Figure 2B). Cell Counting Kit-8 (CCK-8) assay of RAW 264.7 cells shows that the viability of MAs maintains at a high level (> 80%) after incubation with the NGs at a DOX concentration of 20 μg/mL or below, and remains to be 70.4% at the DOX concentration of 40 μg/mL. In contrast, the cell viability sharply declines to < 20% when MAs were directly treated with free DOX at 5 μg/mL. This suggests that the hybrid DOX-loaded NGs have a slow DOX release, which is beneficial for subsequent loading within MAs for tumor delivery. Accordingly, the HA/DOX@PPy NGs with a DOX concentration of 40 μg/mL or below were utilized to examine their loading within MAs.

To investigate the NG loading capability of MAs, we incubated the HA/DOX@PPy NGs with varying DOX concentrations ([DOX] = 10, 20 or 40 μg/mL) with MAs for different time periods (1, 2, 4, 6 or 8 h). After that, the collected MAs-NGs were counted and cracked by the repeated freeze-thawing process to completely release the internalized HA/DOX@PPy NGs before quantification of DOX through UV-vis spectroscopy. As shown in Figure 2C, the intracellular DOX uptake evidently increases to 8.58 pg/cell after 4 h incubation, and slightly increases to 9.03 pg/cell when extending the incubation time to 8 h. Simultaneously, we also show that the DOX uptake amount is concentration-dependent and can reach 11.07 pg/cell at the DOX concentration of 40 μg/mL for the incubated NGs (Figure 2D). The obvious color variation of the collected MAs also verified the successful loading of HA/DOX@PPy NGs (Figure S9). Based on these investigations, we selected the incubation of the HA/DOX@PPy NGs with a DOX concentration at 20 μg/mL for 4 h for the following studies.

Based on the DOX fluorescence in the HA/DOX@PPy NGs, confocal laser scanning microscope was employed to explore the internalization behavior of NGs by the MAs. Unexpectedly, only slight DOX fluorescence can be found in the cytosol of MAs treated with the HA/DOX@PPy NGs for 4 h and 12 h, respectively (Figure S10), which is opposed to the free DOX group. To find out the reason behind, fluorescence spectroscopy was carried out to compare the DOX fluorescence intensity before and after loading within the HA@PPy NGs (Figure S11), free DOX with the same concentration (1 mg/mL) was tested as control. Clearly, loading of DOX within the HA@PPy NGs results in a significant quenching of DOX fluorescence, likely due to the promoted aggregation of DOX after loading within the NGs. This can reasonably explain why the MAs display very weak fluorescence after incubation with the hybrid NGs, in good accordance with the literature 32. The stronger fluorescence signals of MAs after 12 h incubation with the NGs than those after 4 h incubation might be attributed to the increased release of DOX from the HA/DOX@PPy NGs over time.

MAs are reported to express CD44 receptors on the cell surface, which can specifically bind to HA 39, 40. To examine the specific MA uptake of the HA/DOX@PPy NGs through a receptor-mediated manner, flow cytometry analysis of MAs was carried out based on the DOX fluorescence of the HA/DOX@PPy NGs. RAW264.7 cells having both high-level CD44 receptors (RAW-HCD44 cells) and low-level CD44 receptors (RAW-LCD44 cells) were incubated with the hybrid NGs for 4 h before analysis. As shown in Figure S12, compared with the RAW-LCD44 cells pretreated with free HA, RAW-HCD44 cells display a significantly higher DOX fluorescence intensity (p < 0.001) at various DOX concentrations, demonstrating the receptor-mediated uptake of the HA/DOX@PPy NGs by MAs 41, 42.

### Transwell migration and MA phenotype assays

We next checked if the loading of HA/DOX@PPy NGs would affect the intrinsic tumor-homing property and the phenotype of MAs. The chemotactic migration of MAs toward cancer cells was examined by transwell migration assay (Figure 2E-F). As shown in Figure 2F, MAs-NGs display comparable migration percentage (43% *vs* 45%) with NGs-free MAs, both of which can efficiently migrate from the upper chamber to the bottom chamber in the presence of 4T1 cells as indicated by significant amount of cells stained by crystal violet (Figures 2G and 2I). In contrast, in the absence of 4T1 cells in the lower chamber, only few MAs (MAs: 10.2% and MAs-NGs: 9.2%) were observed in Figures 2H and 2J. This implies that the homing property of the MAs-NGs toward cancer cells remains unaffected after loading of the HA/DOX@PPy NGs.

**Figure 3:**
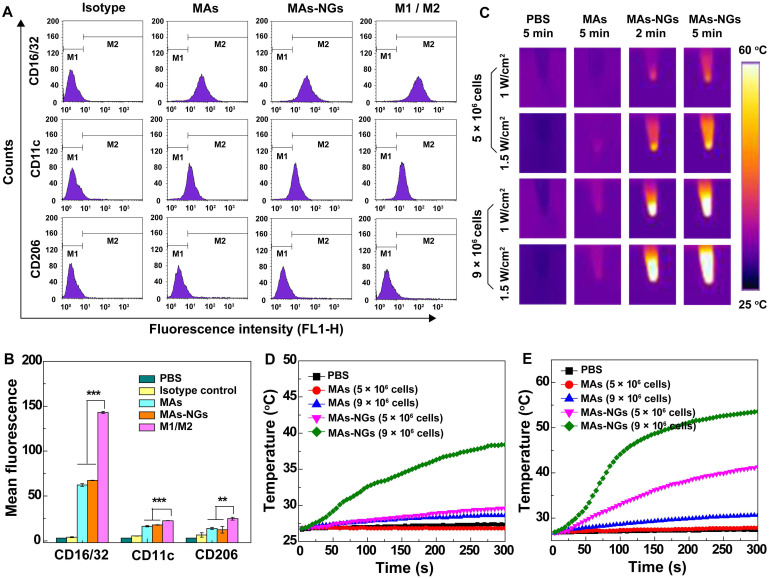
Flow cytometry analysis (A) and corresponding mean fluorescence histograms (B) of the surface markers (M1 phenotype marker: CD16/32 and CD11c; M2 marker: CD206) expression on different RAW264.7cells (MAs, MAs-NGs, LPS-induced M1 MAs, and IL-4-induced M2 MAs). *In vitro* photothermal images (C) and temperature changes of MAs and MAs-NGs with different cell counts (5 × 10^6^ or 9 × 10^6^ cells) when irradiated by an 808-nm laser for 5 min at a power density of 1 W/cm^2^ (D) and 1.5 W/cm^2^ (E). The ** and *** represent p < 0.01 and p < 0.001, respectively.

To examine the impact of the NG loading on the MA phenotype, the expression of MA surface protein markers including CD16/32, CD11c and CD206 were analyzed *via* flow cytometry. MAs are generally classified into classically activated M1 and alternatively activated M2 phenotypes 43. M1-type MAs are considered to be beneficial for tumor suppression, while M2-type MAs are associated with the progression of solid tumors. CD16/32 and CD11c are highly expressed on M1 cell surface, while CD206 tends to be overexpressed on the M2-type MA surface 44, 45. Herein, M1-phenotype MAs stimulated by LPS (1 μg/mL) and M2-type MAs induced by IL-4 (10 ng/mL) were used as controls. According to the flow cytometry assay results in Figure 3A-B, the expression of CD16/32 (67.4 ± 0.21 *vs* 62.3 ± 1.47), CD11c (17.8 ± 0.24 *vs* 16.3 ± 0.16) and CD206 (12.4 ± 3.63 *vs* 13.9 ± 1.24) on the surface of both MAs-NGs and MAs are quite close and no significant changes can be found. Notably, all of the surface marker expressions are much lower than those of the positive controls (M1- or M2-type MAs, p < 0.01). This suggests that the loading of HA/DOX@PPy NGs does not seem to impact the phenotype of MAs.

### *In vitro* photothermal property of MAs-NGs

To examine if the MAs loaded with the HA/DOX@PPy NGs display photothermal properties, the photothermal performance of the MAs-NGs suspension (5 × 10^6^ or 9 × 10^6^ cells, dispersed in 200 μL PBS) was checked after laser irradiation for 300 s (Figure 3C-E). The MAs suspension (5 × 10^6^ or 9 × 10^6^ cells in 200 μL PBS) and PBS solution (200 μL) were used as controls. Clearly, the ΔT value of the MAs-NGs suspension with 9 × 10^6^ cells is 11.6 °C at a power density of 1 W/cm^2^ (Figure 3D), then increases to 26.7 °C at an increased laser power density of 1.5 W/cm^2^ (Figure 3E). Moreover, the ΔT value of MAs-NGs suspension with 5 × 10^6^ cells is merely 3 °C at 1 W/cm^2^ (Figure 3D), while it remarkably increases to 14.6 °C at 1.5 W/cm^2^ (Figure 3E). It reveals that the temperature changes of the MAs-NGs is dependent on cell counts and the laser power density. In contrast to the MAs-NGs, the control groups of MAs and PBS do not exhibit any apparent temperature changes under the same conditions. Simultaneously, the infrared thermal images of corresponding samples under different laser power densities (1 W/cm^2^ or 1.5 W/cm^2^) were also recorded (Figure 3C), displaying photothermal behaviors in consistence with the above results. The superior* in vitro* photothermal performance of the MAs-NGs suggests the successful loading of NGs within the MAs for further tumor therapy applications.

### DOX and NG release from the MAs-NGs and* in vitro* antitumor efficacy of MAs-NGs

**Figure 4:**
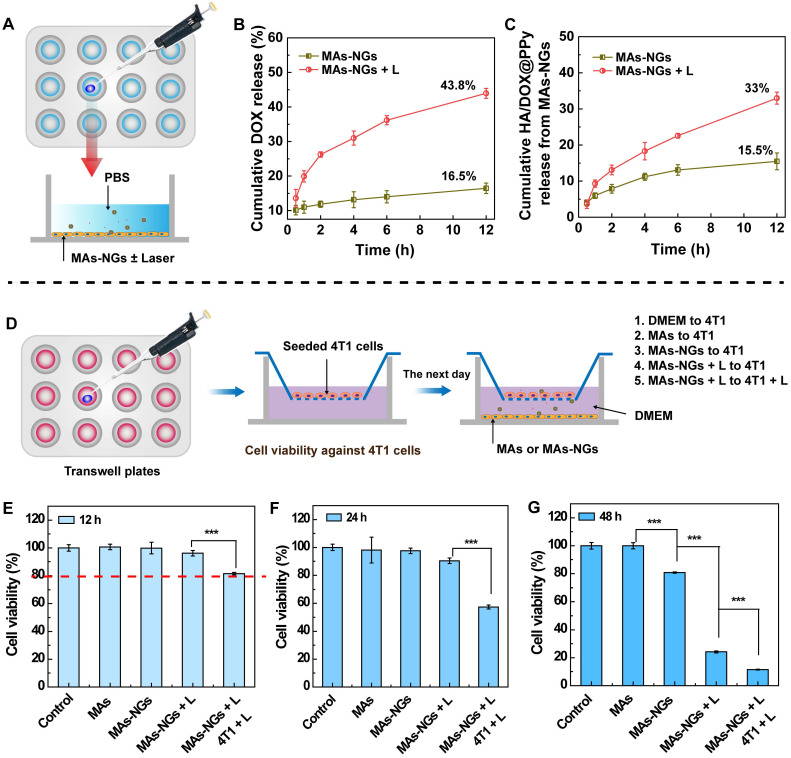
Schematic diagram to test the DOX and NG release from the MAs-NGs. MAs-NGs were incubated with PBS with or without laser irradiation (A). The cumulative release of DOX (B) and HA/DOX@PPy NGs (C) from MAs-NGs. Viability test of 4T1 cells in the transwell system (D). The viability of 4T1 cells tested after treatment for 12 h (E), 24 h (F) and 48 h (G), respectively (*** p < 0.001).

The release of DOX and HA/DOX@PPy from the MAs-NGs in the absence or presence of NIR laser was assessed* in vitro* by recording the UV-vis absorption at 490 and 808 nm, respectively (Figure 4A). As shown in Figure 4B-C, the cumulative release of DOX and HA/DOX@PPy increases with the incubation time, and the applied NIR laser enables enhanced release of both substances. At 12 h, only 16.5% of DOX and 15.5% of HA/DOX@PPy NGs are respectively released from the MAs-NGs in the absence of NIR laser, suggesting that the release rate of both DOX and NGs from the cell carriers is rather limited. However, under an 808-nm laser irradiation for 5 min at 1.5 W/cm^2^, the release of DOX and HA/DOX@PPy NGs greatly increases to 43.8% and 33.0% at 12 h, respectively. The NIR laser-induced fast release might be due to the heat-induced damage of MAs-NGs. We then verified the viability of MAs-NGs with or without NIR laser (Figure S13). A significant cell viability drop can be seen for the MAs-NGs + L group (p < 0.001) when compared to other groups, in accordance with the above assumption. Additionally, we show that the cumulative DOX release is higher than that of the HA/DOX@PPy NGs, which might be attributed to the NIR-promoted additional DOX release from NGs.

Next, the* in vitro* antitumor efficacy of MAs-NGs against 4T1 cells was investigated by CCK-8 assay processed in a transwell system (Figure 4D). The treatments were grouped as follows: Group 1, DMEM to 4T1; Group 2, MAs to 4T1; Group 3, MAs-NGs to 4T1 (Chemotherapy group); Group 4, MAs-NGs + L to 4T1 (NIR-promoted chemotherapy group); and Group 5, MAs-NGs + L to 4T1 + L (NIR-promoted chemotherapy + PTT group). After incubation for 12 h (Figure 4E), the viability of 4T1 cells for the MAs-NGs + L to 4T1 + L group clearly decreases in comparison with other groups, but still higher than 80%. The cell viability significantly drops to 57.4% after treatment for 24 h (Figure 4F). Notably, the 4T1 cell viability in the groups 3-5 are all significantly lower than the control DMEM and MAs groups (p < 0.001) after 48 h treatment (Figure 4G). The *in vitro* antitumor efficacy follows the order of MAs-NGs + L to 4T1 + L > MAs-NGs +L to 4T1 > MAs-NGs to 4T1> MAs to 4T1 > DMEM to 4T1, indicating that NIR can not only promote DOX release from MAs-NGs, but also induce prominent combinational photothermo-chemotherapy.

### *In vivo* MA-mediated delivery of HA/DOX@PPy NGs to tumors

**Figure 5:**
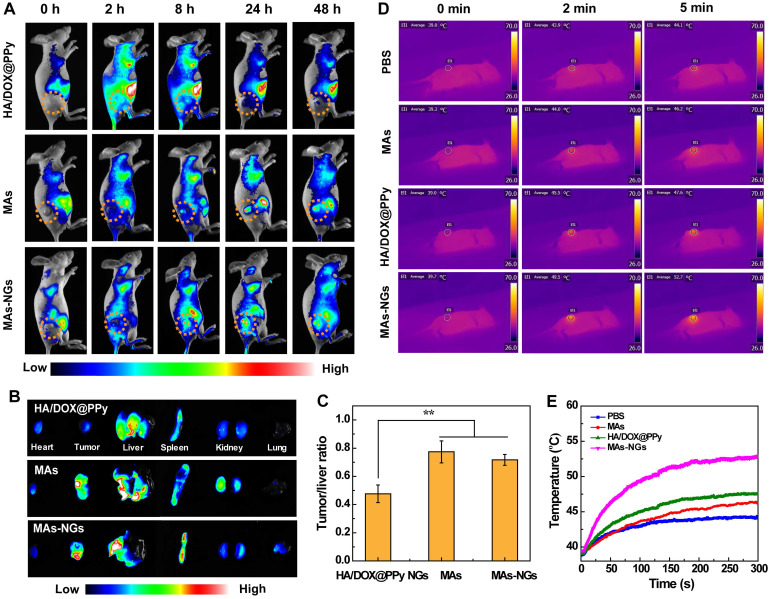
*In vivo* fluorescence images of 4T1-tumor-bearing nude mice at different time points post intravenous injection of HA/DOX@PPy NGs, MAs and MAs-NGs (A). *Ex vivo* fluorescence images of major organs and tumors (B), and the corresponding tumor/liver fluorescence ratio (C) at 48 h post-injection of HA/DOX@PPy, MAs and MAs-NGs, respectively (** p < 0.01). *In vivo* thermal images (D) and temperature changes (E) of tumor-bearing nude mice after different treatments when exposed to an 808 nm laser at 1.5 W/cm^2^ for 5 min.

To confirm the efficient MA-mediated delivery of HA/DOX@PPy NGs to tumors, MAs-NGs were stained by 1,1-dioctadecyl-3,3,3,3-tetramethylindotricarbocyaine (DiR) iodide before intravenous injection to 4T1-bearing tumor mice, and the mice were subjected to whole body fluorescence imaging. The mice treated with Cy7-labeled HA/DOX@PPy NGs were used as control. As shown in Figure 5A, at 2 h post-injection, the tumors treated with the HA/DOX@PPy NGs display the peak Cy7 fluorescence signal and decreased fluorescence intensity at the time point of 8 h and later time points. In sharp contrast, the tumors treated with the MAs or MAs-NGs start to have apparent DiR fluorescence signals at 8 h post-injection, and reach the maximum fluorescence intensity at 24 h post-injection, and the fluorescence signal of tumors can be even maintained until 48 h. The change of tumor fluorescence signals with time post-injection can be further confirmed by quantitative analysis (Figure S14), where MAs and MAs-NGs groups present significantly stronger fluorescence intensity than the Cy7-labeled HA/DOX@PPy NGs group at 24 and 48 h post administration (p < 0.001). This suggests that with the MA-mediated delivery and tumor homing, the NGs can be maintained within the tumor region with an extended time period, which is beneficial for further improved tumor therapy.

Furthermore, we euthanized the mice at 48 h post-injection and collected their major organs and tumors for *ex vivo* fluorescence imaging to investigate the biodistribution behaviors of NGs, MAs and MAs-NGs (Figure 5B-C). Notably, the MAs-NGs retain the comparable tumor targeting ability with NG-free MAs, and both MAs and MAs-NGs are mainly distributed in the tumor, liver and spleen. At the same time point, the tumor fluorescence intensity of the NGs groups is much lower than those of the MAs and MAs-NGs groups (Figure S15), although it is a bit unfair to make such a comparison because different dyes and different labelling subjects (NGs versus cell membranes) were compared. To be in a more actual way, we analyzed the fluorescence ratio of tumor/liver to check the difference in groups (Figure 5C). Clearly, the tumor/liver fluorescence ratios in the groups of MAs and MAs-NGs are much higher than the NGs group (p < 0.01). This also suggests that with the MA-mediated tumor homing, the NGs can readily escape the reticuloendothelial system for improved tumor treatment.

### *In vivo* photothermal imaging of tumors

*In vivo* photothermal imaging of 4T1 tumor-bearing nude mice after different treatments (PBS, MAs, HA/DOX@PPy NGs or MAs-NGs) were next investigated (Figure 5D-E). As can be seen, the injection of MAs-NGs leads to the most significant thermal contrast among all groups, indicating the best tumor homing ability of the NGs mediated by MAs. The temperature of tumor site in mice treated with the MAs-NGs increases by 13 °C under an 808-nm laser irradiation (1.5 W/cm^2^) for 5 min, while the tumor temperature of mice treated with PBS and MAs only increases slightly (5.1 and 6.9 °C, respectively). Moreover, the tumor temperature change of the mice directly injected with the HA/DOX@PPy NGs is 8.6 °C, also much lower than the MAs-NGs group, which is due to the lack of active tumor homing.

### *In vivo* therapeutic efficacy of 4T1 tumors

**Figure 6:**
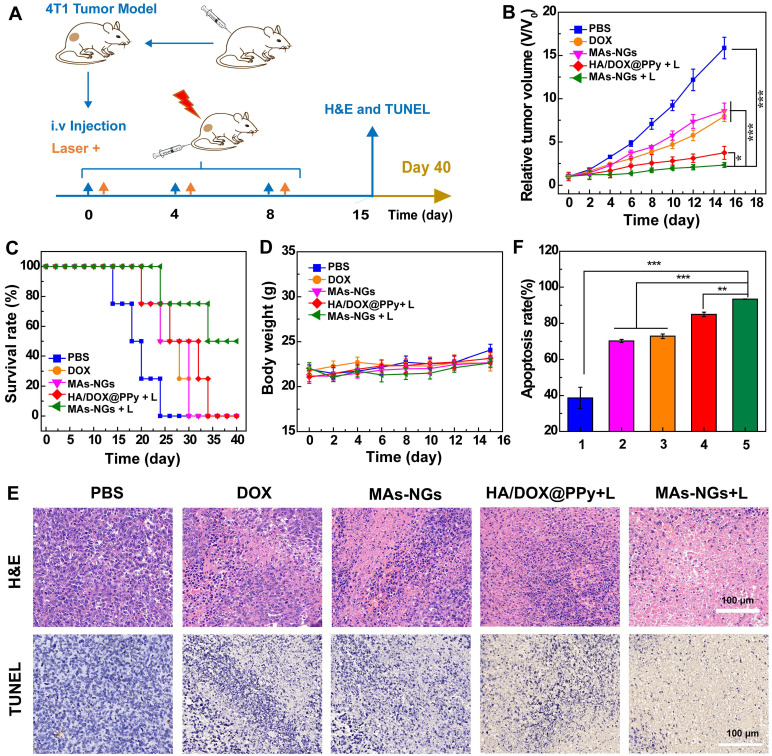
*In vivo* therapeutic process (A), relative tumor volume (B), survival rate (C) and body weight changes (D) of 4T1-tumor bearing nude mice after different treatments (n = 5). H&E and TUNEL staining analysis of tumor slices (E) on day 15 post-treatment. Quantitative analysis of the apoptosis rate (F) of tumor cells corresponding to TUNEL images determined by Image J software. 1-5 represents the groups of PBS, MAs-NGs, free DOX, HA/DOX@PPy NGs + Laser, and MAs-NGs + Laser, respectively.

Based on their intrinsic phagocytic and tumor-homing properties, MAs have been considered to be an effective biocarrier for targeted drug delivery to solid tumors 41, 46. To verify the exerted therapeutic effect of the HA/DOX@PPy NGs through MA-mediated tumor delivery, a subcutaneous 4T1 tumor model was established and received combination PTT/chemotherapy (Figure 6A). The injected quantity of DOX for groups of free DOX, HA/DOX@PPy NGs and MAs-NGs were kept consistent for reasonable comparison. As shown in Figure 6B, the relative tumor volume (V/V_0_) data reveals that the treatment with HA/DOX@PPy + Laser or MAs-NGs + Laser for two weeks leads to a significant tumor suppression when compared to other groups (p < 0.001), and the tumor growth inhibition rate in the group of MAs-NGs + Laser is significantly higher than that in the group of HA/DOX@PPy NGs + Laser (p < 0.05), clarifying the role played by MA-mediated tumor homing to have extended tumor retention of the therapeutics. On the contrary, the mice in PBS group exhibit the most rapid tumor growth, and the tumor progression remains relatively fast although a certain tumor inhibition effect was found in the free DOX and MAs-NGs group. It is interesting to note that under the same conditions, the combination PTT/chemotherapy group (MAs-NGs + Laser) exhibits more significant therapeutic effect than the single chemotherapy group (MAs-NGs), which might be due to the NIR laser-promoted drug/NG release from the MAs-NGs for efficient uptake by tumor cells and the NIR laser-generated heat to destroy tumor cells as well. Further, the slightly acidic tumor microenvironment also facilitates the fast DOX release to enhance its chemotherapy effect. The triggered fast drug release in tumor region is beneficial to reduce the toxic effects of drugs on normal organs/tissues. The tumor inhibition efficacy follows the order of MAs-NGs + Laser > HA/DOX@PPy NGs + Laser > free DOX > MAs-NGs > PBS.

Further survival rate analysis reveals that mice in the MAs-NGs + laser group have the highest survival rate (50% survival) until 40^th^ day post-treatment, while all mice died at 34, 30, 30, and 24 days for the HA/DOX@PPy NGs + Laser, free DOX, MAs-NGs and PBS groups, respectively (Figure 6C). This further verifies the best antitumor efficacy of the MAs-NGs + Laser group that enables MA-mediated tumor delivery of the hybrid NGs for combination PTT/chemotherapy. Simultaneously, the body weights of mice in all groups were recorded (Figure 6D), and no obvious body weight changes can be observed for all groups during the whole treatment process. This means that all treatments do not seem to generate toxic effects to mice. The non-toxic effect of all treatment groups was further confirmed through hematoxylin & eosin (H&E) staining of the major organs of mice on the 15th day post-treatment (Figure S16). No considerable abnormality can be observed in the stained slices of the heart, liver, spleen, lung and kidney for all treatment groups, in accordance to the PBS control group. It is worthwhile to noting that the non-toxic effect of free DOX should be due to the relatedly low dose injected as compared to the literature 47.

To further check the treatment efficacy of MAs-NGs + Laser, H&E and terminal deoxynucleotidyl transferase-mediated dUTP-biotin nick end labeling (TUNEL) staining of tumor tissues were performed (Figure 6E-F). As opposed to the PBS control, a large number of necrotic and apoptotic tumor cells can be seen in the MAs-NGs + laser group, followed by the groups of HA/DOX@PPy + Laser, free DOX, and MAs-NGs. The tumor cell apoptosis rate was further quantified based on the TUNEL staining images (Figure 6F). Clearly, the treatment of the MAs-NGs + laser leads to a significantly higher apoptosis rate than other groups (p < 0.01), and the apoptosis rate follows the exactly same order of the tumor inhibition rate (Figure 6B).

The biosafety of exogenous RAW264.7 cells on healthy nude mice were also evaluated on day 7 post treatment with MAs or MAs-NGs, and the PBS-treated mice were used as control. The key biomarkers in serum such as alanine aminotransferase (ALT), aspartate aminotransferase (AST), blood urea nitrogen (BUN) and creatinine (CREA) used to assess the liver and kidney damage were analyzed. As shown in Figure S17A-D, no significant changes are observed for different groups when compared to the PBS control. This suggests that the MAs or MAs-NGs have no significant toxicity to the liver and kidney. Moreover, the serum level of inflammatory cytokine interleukin-6 (IL-6) was also measured according to the literature 48, 49 to assess whether the exogenous RAW264.7 cells can induce undesirable immune response. The results shown in Figure S17E reveal that the treatment of MAs or MAs-NGs does not significantly impact the IL-6 secretion, meaning that the exogenous RAW264.7 cells do not cause any systemic immunotoxicity *in vivo*. Simultaneously, the slight increase in pro-inflammatory cytokines of IL-12, tumor necrosis factor-alpha (TNF-α) and interferon gamma (IFN-γ) (Figure S17F-H) might be due to the partial polarization of MAs to antitumor M1-type 50.

## Conclusions

In summary, we present an updated design of MA-mediated delivery platform of HA/DOX@PPy NGs for enhanced tumor combination PTT/chemotherapy. We show that Cys-crosslinked HA NGs prepared through a double emulsion method can be loaded with both a photothermal agent of PPy and anticancer drug DOX with good colloidal stability, and display an enhanced loading within MAs through their surface CD44 receptor-mediated endocytosis. The MAs loaded with the hybrid NGs under optimal concentrations display no appreciable phenotype changes and non-compromised tumor homing property largely due to the strong coordination between the PPy and the DOX drug to avoid fast DOX release. With the MA-mediated tumor homing, the hybrid NGs with a good PPy-induced photothermal conversion efficiency (52.7%) enable fast DOX/NG release at the tumor site under laser irradiation, thereby facilitating enhanced combination PTT/chemotherapy to significantly inhibit the tumor growth. The developed method to use MA-mediated tumor homing of hybrid NGs may be extended to combine different therapeutic and imaging elements for different cancer nanomedicine applications.

## Experimental Section

### Synthesis of HA NGs

HA NGs were prepared using a double-emulsion method according to literature protocols with minor modifications 28, 51. In brief, 1-ethyl-3-[3-(dimethylamino)propyl] carbodiimide hydrochloride (EDC, 4.75 mg) was added to 2 mL of sodium hyaluronate solution (20 mg, 1 wt%) to activate the carboxyl groups of HA under continuous stirring for 3 h. The activated HA aqueous solution was then dropwise added to dioctylsodium sulfosuccinate (2.5 wt%, 4 mL) pre-dissolved in dichloromethane (DCM) under stirring for 10 min to achieve a water-in-oil (w/o) emulsion. Afterwards, the w/o emulsion was slowly dropped into a poly(vinyl alcohol) aqueous solution (2 wt%, 30 mL) under vigorous stirring for 15 min to get the w/o/w double emulsion. Subsequently, Cys (22.33 mg, dissolved in 1 mL water) was rapidly added into the above mixture under continuous stirring overnight to remove DCM completely. Followed by dialysis against water (6 times, 2 L) using a regenerated cellulose dialysis membrane (molecular weight cut-off (MWCO) = 1,000,000) for 3 days and centrifugation at 13000 rpm for 5 min, the purified HA NGs were finally acquired. Moreover, here we chose sodium hyaluronate with 3 different molecular weights (Mw = 48, 320 and 950 kD, respectively) to synthesize HA NGs in order to investigate the effect of HA Mw on the formation of NGs. A fraction of the purified HA NGs was subjected to lyophilization to determine the mass concentration and the remaining was stored at 4 °C for further use.

### Preparation of HA@PPy NGs

PPy NPs were incorporated within HA NGs through an* in-situ* oxidation polymerization reaction 24, 52. In brief, an HA NG solution (86 mg, 5 mL in water) was added with ferric trichloride hexahydrate (FeCl_3_. 6H_2_O) according to the ratio of its mass to the volume of Py monomer at 9 mg : 1 μL under stirring for 1 h to form an iron-red mixture solution. The mixture was then transferred into an ice bath condition and added with different amounts of Py monomers (with mass ratio of HA NGs/Py at 1: 0.11, 1: 0.23, 1: 0.46, 1: 0.69, or 1: 0.92). Under an ice-bath condition, oxidation polymerization was performed for 4 h. Then, the reaction mixture was dialyzed against water (2 L, 6 times) using dialysis membranes (MWCO = 14,000) for 3 days to obtain the aqueous suspension of HA@PPy NGs.

### Loading of DOX within the HA@PPy NGs

DOX was physically encapsulated within the HA@PPy NGs through π-π stacking and hydrophobic interaction with PPy NPs. In brief, 2.5 mg of DOX∙HCl was dispersed in methanol, deprotonated using triethylamine (300 μL), and added into the aqueous HA@PPy NGs solution (10 mg, 5 mL) for 12 h under stirring overnight to evaporate the methanol. Followed by centrifugation at 5000 rpm for 10 min to remove the un-complexed precipitated DOX, the supernatant was collected, centrifuged at 13000 rpm for 15 min, and washed 2 times with water to get the final HA/DOX@PPy NGs solution. The precipitated DOX was also collected, re-dissolved in 1 mL of methanol, and quantified through UV-vis spectroscopy.

### Loading of HA/DOX@PPy NGs within MAs

RAW 264.7, a murine macrophage cell line, were seeded into 12-well plates at a density of 1 × 10^5^ cells per well and cultured in complete Dulbecco's modified Eagle's medium (DMEM) supplemented with 10% FBS, 1% streptomycin and penicillin overnight to achieve about 70% confluence. The next day, the cells were incubated with fresh DMEM containing the HA/DOX@PPy NGs (10, 20 or 40 μg/mL of DOX) for different time periods (1, 2, 4, 6 or 8 h) to obtain the MAs loaded with the HA/DOX@PPy NGs (MAs-NGs).

### *In vitro* cell biological assays

*In vitro* cytotoxicity, cellular uptake, transwell cell migration, and cell phenotype assays were performed after MAs were incubated with the HA/DOX@PPy NGs. In addition, the heat generation and thermal imaging property of MAs loaded with the HA/DOX@PPy NGs were also investigated. See additional experimental details in the Supporting Information.

### *In vivo* animal studies

All animal experiments were carried out after approval by the Animal Care and Use Committee (IACUC) of Donghua University and also following the policy of the National Ministry of Health. A subcutaneous 4T1 (a murine breast cancer cell line) tumor model was established and used for *in vivo* imaging and therapy studies. See additional experimental details in the Supporting Information.

## Supplementary Material

Supplementary experimental section, figures and tables.
