# Supplementary material for: Macrophage-mediated tumor homing of hyaluronic acid nanogels loaded with polypyrrole and anticancer drug for targeted combinational photothermo-chemotherapy
Source: Theranostics. 2021 May 13;11(14):7057–71. doi: 10.7150/thno.60427 (PMC8171075; doi:10.7150/thno.60427)
Supplement: Supplementary file 1 — Supplementary experimental section, figures and tables. [file thnov11p7057s1.pdf]

Supporting Information

Macrophage-mediated tumor homing of hyaluronic acid nanogels loaded with polypyrrole and anticancer drug for targeted combinational photothermo-chemotherapy

Tingting Xiao, Wei Hu, Yu Fan, Mingwu Shen, Xiangyang Shi*

State Key Laboratory for Modification of Chemical Fibers and Polymer Materials, Shanghai Engineering Research Center of Nano-Biomaterials and Regenerative Medicine, College of Chemistry, Chemical Engineering and Biotechnology, Donghua University, Shanghai 201620, People's Republic of China

Keywords: Macrophages; hyaluronic acid nanogels; polypyrrole; tumor homing property; photothermo-chemotherapy

---

* Corresponding author. E-mail addresses: xshi@dhu.edu.cn

Experimental section

Materials. Sodium hyaluronate (Mw = 48, 320, or 950 kD) was purchased from Bloomage Biotechnology Corp., Ltd. (Jinan, China). Dioctylsodium sulfosuccinate, poly(vinyl alcohol) (87-89% hydrolyzed, Mw = 85-124 kD) and ferric trichloride hexahydrate ($FeCl_3 \cdot 6H_2O$) were obtained from Sinopharm Chemical Reagent Co., Ltd. (Beijing, China). Pyrrole (Py, 98%) was acquired from Sigma-Aldrich (St. Louis, MO). Cystamine dihydrochloride (Cys, 99%) was supplied by Energy Chemical Co., Ltd. (Shanghai, China).1-Ethyl-3-[3-dimethylaminopropyl] carbodiimide hydrochloride (EDC) was from J&K Chemical, Ltd. (Shanghai, China). Dichloromethane was from Shanghai Lingfeng Chemical Reagent Co., Ltd. (Shanghai, China). Doxorubicin hydrochloride (DOX·HCl) was from Beijing Huafeng Pharmaceutical Co., Ltd. (Beijing, China). 1,1-Dioctadecyl-3,3,3,3-tetramethylindotricarbocyaine iodide (DiR Iodide) was from MesGen biotechnology (Shanghai, China). 4',6-Diamidino-2-phenylindole (DAPI) was acquired from BestBio Science (Shanghai, China). All chemicals were used without further purification. Macrophages (for short, MAs, RAW264.7, a murine MA cell line) and a murine breast cancer cell line (4T1 cells) were obtained from Institute of Biochemistry and Cell Biology, the Chinese Academy of Sciences (Shanghai, China). Cell Counting Kit-8 (CCK-8) and interferon gamma (IFN-γ) ELLSA kit were from Beyotime (Shanghai, China). Dulbecco's modified Eagle's medium (DMEM) and fetal bovine serum (FBS) were acquired from Gibco Life Technologies Co. (Grand Island, NY). Penicillin-streptomycin and trypsin were from HyClone Lab., Inc. (Logan, UT). Anti-CD16/32-FITC (fluorescein isothiocyanate), anti-CD11c-FITC and IgG isotype controls were from Thermo Fisher Scientific (Waltham, MA). Anti-CD206 Alexa Fluor® 488 and its IgG isotype were bought from Biolegend, Inc. (San Diego, CA). Alanine transaminase (ALT), aspartate transaminase (AST), urea nitrogen (BUN) and creatinine (CREA) kits were bought from Rayto (Shenzhen, China). Interleukin-6 (IL-6), IL-12 and tumor necrosis factor-alpha (TNF-α) kits were purchased from Multisciences (Hangzhou, China). Regenerated cellulose dialysis membranes with a molecular weight cut-off (MWCO) of 14000 or 1000 000 were acquired

from Fisher (Pittsburgh, PA). Water used in all experiments was purified by a RephiQuatro U Pack 1 water purification system (Rephile Bioscience, Ltd., Shanghai, China) with a resistivity higher than 18.2 MΩ.cm.

Loading of DOX within HA@PPy NGs. DOX was physically encapsulated within the HA@PPy NGs through π-π stacking and hydrophobic interaction with PPy NPs. In brief, 2.5 mg of DOX·HCl was dispersed in methanol, deprotonated using triethylamine (300 μL), and added into the aqueous HA@PPy NGs solution (10 mg, 5 mL) for 12 h under stirring overnight to evaporate the methanol. Followed by centrifugation at 5000 rpm for 10 min to remove the un-complexed precipitated DOX, the supernatant was collected to get the final HA/DOX@PPy NGs in aqueous solution. The precipitated DOX was also collected, re-dissolved in 1 mL of methanol, and quantified through UV-vis spectroscopic measurement of its absorption at 490 nm according to the standard DOX absorbance/concentration calibration curve ($y = 22.5507x + 0.00692$, $R^2 = 0.9991$, with an NG concentration ranging from 0.0025 to 0.04 mg/mL). The entrapment efficiency (EE, w/w%) and loading content (LC, w/w%) of DOX were calculated from the equations of EE_{DOX} = (weight of loaded DOX/initial weight of DOX) × 100% and LC_{DOX} = (weight of loaded DOX/weight of HA/DOX@PPy) × 100%, respectively.

General characterization techniques. Dynamic light scattering (DLS) and zeta potential measurements were performed using a Malvern Zetasizer Nano ZS model ZEN3600 (Worcestershire, UK) equipped with a standard 633-nm laser. UV-vis spectra were collected using a Lambda 25 UV-vis spectrophotometer (Perkin-Elmer, Boston, MA). Samples were dissolved in water with a concentration of 1 mg/mL before measurements. The morphology and size distribution of the prepared HA@PPy NGs were observed by S-4800 scanning electron microscope (SEM, Hitachi, Ltd., Tokyo, Japan) at a voltage of 15 kV and JEM-2100F transmission electron microscopy (TEM, JEOL Ltd., Tokyo, Japan) with an accelerating voltage of 200 kV, respectively. For SEM imaging, a typical sample was prepared by dropping an NG water solution (1 mg/mL, 10 μL) onto aluminum foil, followed by air drying and sputter coating of a gold film with a thickness of 10 nm before measurements. For TEM

imaging, a typical sample of HA@PPy NGs in aqueous solution (1 mg/mL, 10 μL) was deposited onto a carbon-coated copper grid and air-dried before measurements. The particle size distribution was calculated using Image J 1.40 G software (http://rsb.info.nih.gov/ij/download.html). For each sample, at least 300 particles from different SEM or TEM images were randomly selected and analyzed. Fourier transform infrared (FTIR) spectra were collected using a Nexus 670 spectrophotometer (Thermo Nicolet Corporation, Madison, WI) with an attenuated total reflectance (ATR) technique. Solid samples were mixed with KBr crystals, grinded fully and tableted before measurements. Thermal gravimetric analysis (TGA) was carried out using a TG 209 F1 thermo gravimetric analyzer (NETZSCH Instruments Co., Ltd., Selb/Bavaria, Germany) at a heating rate of 10 °C/min under N_2 atmosphere with a temperature ranging from 50 to 800 °C.

Photothermal conversion property. To evaluate the photothermal conversion property of the HA@PPy NGs, the temperature changes of the NG solution at different concentrations (0, 0.125, 0.5, 1 and 2 mg/mL, respectively) were recorded in real-time when exposed to an 808-nm laser device (Shanghai Xilong Optoelectronics Technology Co. Ltd., Shanghai, China). Typically, 0.3 mL of an aqueous HA@PPy NG solution at different concentrations was laser irradiated for 5 min under an output power density of 1.0 W/cm^2. The real-time temperature changes of corresponding samples were recorded every 5 s by an online DT-8891E thermocouple thermometer (Shenzhen Everbest Machinery Industry Co., Ltd., Shenzhen, China). To quantify the photothermal conversion property of the HA@PPy NGs, a heating and cooling cycle of the sample was carried out. The photothermal conversion efficiency (η) of the HA@PPy NGs was calculated according to the literature protocols [1, 2]. Furthermore, to evaluate the photothermal stability of the NGs, 5 heating and cooling (laser on-off) cycles were performed to record the temperature changes.

DOX release from NGs. To investigate the DOX release from the HA/DOX@PPy NGs under different pHs with or without NIR laser irradiation, the HA/DOX@PPy NGs dispersed in phosphate buffered saline (1 mg/mL, pH 7.4) or sodium citrate buffer (pH 5.5 with or without laser) were placed in a regenerated cellulose dialysis bag with an MWCO of 10,000 to have a volume of 1 mL, submerged

into 9 mL of the corresponding buffer medium at 37 °C and kept shaking constantly. For the pH 5.5 + Laser group, the NG solution in the dialysis bag was irradiated for 10 min with an 808-nm laser at a power density of 1 W/cm^2 before the dialysis experiment. At each scheduled time interval, 1 mL of the outer phase buffer medium was taken out and subsequently supplemented with 1 mL of the corresponding buffer to maintain the constant volume of outer phase buffer medium. The DOX absorption at 490 nm was recorded by UV-vis spectrometry to determine the concentration of released DOX according to the corresponding absorption-concentration calibration curve at different pHs.

Cell culture. 4T1 and murine macrophage (RAW264.7) cells were respectively cultured in DMEM supplemented with 10% FBS and 1% penicillin-streptomycin. Particularly, RAW264.7 cells grown in hyaluronic acid (HA)-free medium overexpressed CD44 receptors (denoted as RAW-HCD44 cells), while RAW264.7 cells pretreated in HA-containing medium (2.0 mM HA) expressed low-level CD44 receptors (denoted as RAW-LCD44 cells). The term of MAs or RAW264.7 cells always represented the RAW-HCD44 cells unless otherwise stated specifically.

***In vitro* cytotoxicity to MAs.** The conventional Cell Counting Kit-8 (CCK-8) assay was utilized to evaluate the *in vitro* cytotoxicity of the HA/DOX@PPy NGs to MAs. RAW264.7 cells were seeded into 96-well plates at a density of 1×10^4 cells per well and incubated overnight at 37 °C and 5% CO_2. Afterwards, the medium in each well was replaced with fresh medium (100 μL) containing free DOX or HA/DOX@PPy NGs at different DOX concentrations (5, 10, 15, 20, 30, 40 and 80 μg/mL, respectively), and the cells were continuously cultured for another 24 h. Then, the cells were rinsed with PBS for three times, incubated with 100 μL of FBS-free DMEM supplemented with 10% CCK-8 regent in each well for 3 h. The absorbance of each well at 450 nm was recorded by the Multiskan MK3 microplate reader (Thermo Scientific, Waltham, MA).

***In vitro* MA loading of functional NGs.** The MA loading of HA/DOX@PPy NGs under different incubation time periods (0, 1, 2, 4, 6, or 8 h) and different DOX concentrations (0, 10, 20, or 40 μg/mL) were analyzed by UV-vis spectrometry to record the DOX absorption at 490 nm. In brief, RAW264.7 cells were seeded into 12-well plates (1×10^5 cells per well in 1 mL of complete DMEM containing

10% FBS and 1% penicillin-streptomycin) and cultured overnight. Then, for the following loading of NGs within the macrophage cells, FBS-free DMEM was used for cell culture. For time-dependent loading analysis, the medium in each well was removed, gently washed with PBS, and replaced with fresh FBS-free medium containing the HA/DOX@PPy NGs ([DOX] = 20 μg/mL). The cells were incubated regularly for 1, 2, 4, 6 and 8 h, respectively.

For the concentration-dependent macrophage loading, fresh medium containing the NGs with different DOX concentrations (10, 20 or 40 μg/mL) was utilized to treat the RAW264.7 cells for 4 h. After that, the cells in each well were washed with PBS for 3 times, collected, counted and lysed by 3 cycles of freeze-thawing process in liquid nitrogen. The released DOX was measured by UV-vis spectrometry as described above and the loaded DOX content per cell was calculated by subtracting the background absorption of cells treated with PBS.

Furthermore, the uptake of HA/DOX@PPy NGs by MAs was validated by Confocal laser scanning microscope (CLSM, ZEISS LSM 700, Jena, Germany) through observation of the DOX fluorescence. Briefly, 1×10^5 RAW264.7 cells were seeded into each confocal dish and cultured overnight at 37 °C and 5% CO_2. After that, the HA/DOX@PPy NGs with a DOX concentration of 20 μg/mL were chosen to treat the cells for 4 h and 12 h, respectively. Free DOX and PBS were used as the positive and negative controls, respectively. DAPI was used to stain the MA nuclei before observation according to the standard protocols reported in our previous work [3].

Cell migration assay. Cell migration assay through a transwell system was used to investigate whether the NG-loaded MAs (MAs-HA/DOX@PPy NGs, for short, MAs-NGs) remained their tumor-homing property *in vitro*. MAs without NGs were used as control. The transwell system was equipped with transwell inserts supported by polycarbonate membranes (Corning Incorporated, Corning, NY) with a pore size of 8.0 μm, and fitted into the lower chamber of a 24-well cell culture plate. 4T1 cells were seeded in the lower chamber at a density of 5×10^4 cells/well in 0.5 mL of DMEM in advance, and cultured overnight at 37 °C and 5% CO_2. Blank DMEM without 4T1 cells was also placed in the

lower chamber in parallel and used as the negative control. The next day, 200 μL of MAs-NGs or MAs suspension was seeded in the upper chamber (2×10^4 cells in FBS-free DMEM for each insert), and co-incubated continuously for another 18 h to accomplish the transmigration process from the upper to lower chamber. Afterwards, medium of each insert was removed, and cells in each insert were gently washed with PBS for 2 times, fixed with formaldehyde (1 mL, 4% in PBS) for 15 min, washed with PBS for 2 times again, and stained with crystal violet (0.1%, in 1 mL PBS) for 30 min. Then, we gently washed away the surplus crystal violet using PBS (2 ~ 3 times) and carefully wiped out those unmigrated cells using a wet cotton swab. The stained MAs-NGs or MAs transmigrated to the bottom side of the transwell inserts were observed by phase contrast microscopy. At least 20 images were randomly selected for cell counting and statistical analysis for each sample.

Evaluation of MA phenotype change. The MA surface protein marker expression after uptake of HA/DOX@PPy NGs was examined by Becton Dickinson FACScan flow cytometer (FACS, Franklin Lakes, NJ). Briefly, RAW264.7 cells were seeded into 12-well plates at a density of 2×10^5 cells per well with 1 mL DMEM. For comparison, M1- and M2-type of MAs were also created by treatment of each well of cells with 1 μg/mL lipopolysaccharide (LPS) and 10 ng/mL IL-4, respectively for 24 h. After that, the culture medium in each well was removed and supplemented with 1 mL of fresh medium containing the HA/DOX@PPy NGs ([DOX] = 20 μg/mL) for MAs-NGs group, or 1 mL of blank medium for the control groups of M1/M2 MAs and the negative control group of MAs treated with PBS. The cells of each well were continuously cultured for 4 h, harvested, and incubated with 5 μL of the rat monoclonal antibodies of CD16/32 -FITC, CD11c-FITC and CD206 Alexa Fluor® 488, respectively for 30 min under an ice bath condition before flow cytometry analysis. The corresponding isotype controls were also treated according to the above protocols.

***In vitro* thermal imaging of MAs-NGs.** To explore the *in vitro* photothermal property of HA/DOX@PPy NGs after they were taken up by MAs (MAs-NGs), thermal imaging of the MAs-NGs suspension at different concentrations (with 5×10^6 or 9×10^6 MAs-NGs dispersed in 0.3 mL of PBS) was performed after exposed to an 808-nm laser at an output power density of 1 W/cm^2 or 1.5 W/cm^2.

The thermal images were recorded by an FLIR infrared thermal camera (IRS Systems Inc., Shanghai, China). In brief, MAs in Corning^{TM} cell culture flasks (T-25, *ca.* 9.0 × 10^6 cells/flask) were incubated with 5 mL DMEM containing the HA/DOX@PPy NGs ([DOX] = 20 μg/mL) for 4 h. MAs treated with PBS were used as control. Then, the MAs-NGs or MAs in each flask were washed with PBS for 3 times, trypsinized and redispersed in a 0.5-mL Eppendorf tube before thermal imaging. Simultaneously, the real-time temperature variations of each sample with different cell suspension concentrations and different laser power densities were also recorded by the online DT-8891E thermocouple thermometer (Shenzhen Everbest Machinery Industry Co., Ltd., Shenzhen, China).

DOX and NG release from the MAs-NGs and the *in vitro* antitumor efficacy of MAs-NGs. In order to evaluate the DOX and NG release from the MAs-NGs, MAs (2×10^5 cells/well) were seeded in 12-well plates and incubated at 37 ºC and 5% CO_2 overnight. The next day, the cells were treated with HA/DOX@PPy NGs ([DOX] = 20 μg/mL) for 4 h and washed twice with PBS to obtain the MAs-NGs. Extra 3 parallel wells were taken to count the number of cells laden with MAs-NGs. Afterwards, PBS (1 mL/well) instead of medium was added in each well and the cells were continuously incubated. Notably, in the MAs-NGs + L group, the cells were irradiated with an 808-nm laser at a power density of 1.5 W/cm^2 for 5 min. Then, at a specific time interval (0.5, 1, 2, 4, 6 and 12 h, respectively), 1 mL of supernatant was collected for UV-vis spectrometric analysis and the cells were supplemented with 1 mL of PBS. The released DOX and HA/DOX@PPy NGs were determined by UV-vis spectral analysis at 490 and 808 nm, respectively using the respective standard concentration-absorption calibration curve.

To assess the *in-vitro* therapeutic activity of the MAs-NGs against 4T1 cells in the absence or presence of an NIR laser, a 12-well transwell system was employed. Firstly, 4T1 cells (2.5×10^4 cells/well) were seeded in the upper chamber and incubated with complete DMEM under regular cell culture conditions. MAs-NGs were collected after pretreatment with the HA/DOX@PPy NGs ([DOX] = 20 μg/mL) for 4 h, and seeded in the lower chamber of the transwell system with a density of 1×10^6

cells per well, and then the upper chamber was merged with the lower chamber and cells were co-incubated for different time periods (12, 24 and 48 h, respectively). For the MAs + L to 4T1 group and MAs + L to 4T1 + L group, MAs or/and 4T1 cells were irradiated with an 808-nm laser at a power density of 1.5 W/cm^2 for 5 min. DMEM and MAs group were used as controls. At specific time intervals, the upper chamber was inserted into a new 12-well plate, and the 4T1 cells were rinsed with PBS for three times, added with 100 μL/well of FBS-free DMEM supplemented with 10% CCK-8 regent and incubated for another 3 h. Then, we transferred the solution from upper chamber to 96-well plates and the absorbance of each well at 450 nm was recorded by the Multiskan MK3 microplate reader.

Animal models. All animal experiments were carried out after approval by the Animal Care and Use Committee (IACUC) of Donghua University and also following the policy of the National Ministry of Health. The subcutaneous 4T1 tumor model was established for *in vivo* imaging and therapy studies. Female nude mice, 4-6 week old, were purchased from Shanghai Slac Laboratory Animal Co., Ltd. (Shanghai, China). Each mouse was subcutaneously inoculated with 2 × 10^6 4T1 cells (100 μL in PBS) onto its right back. When the tumor volume reached 80-100 mm^3 after inoculation for 7-10 days, the tumor models were ready for the following *in vivo* imaging and therapy experiments.

***In vivo* and *ex vivo* fluorescence imaging.** To confirm the *in-vivo* tumor-homing property of MAs and MAs-NGs, both MAs and MAs-NGs were stained by a cell membrane dye of 1,1-dioctadecyl-3,3,3,3-tetramethylindotricarbocyaine (DiR) iodide before injection. The fluorescence imaging of the tumor-bearing mice at different time intervals (0, 2, 8, 24 and 48 h, respectively) before and after intravenous administration of MAs-NGs or MAs (5 × 10^6 cells, in 100 μL PBS) was carried out using an *in-vivo* fluorescence imaging system (IVIS) with a CCD camera (Tanon ABL X6 In Vivo Imaging System, Shanghai, China). HA/DOX@PPy NGs pre-labeled with Cyanine 7 amine (Cy7-NH_2) (2.23 mg/mL, [DOX] = 429 μg/mL, in 100 μL PBS) were also injected for comparison. Each mouse was anesthetized with pentobarbital sodium (40 mg/kg) *via* intraperitoneal injection before

injection. To investigate the fluorescence signal biodistribution after different treatments, the mice were euthanized at 48 h after IVIS imaging, and major organs (heart, liver, spleen, lung, and kidney) and tumor tissues were extracted for *ex vivo* fluorescence imaging. All fluorescent imaging data at the regions of interest (ROI) were acquired and analyzed using the IVIS imaging software.

***In vivo* tumor thermal imaging.** The *in vivo* photothermal imaging performance of the MAs-NGs was validated using a subcutaneous tumor model through an infrared camera. In brief, 5×10^6 MAs were collected, redispersed in 100 μL of PBS after incubation with the HA/DOX@PPy NGs ([DOX] = 20 μg/mL) for 4 h, and then intravenously (*i.v.*) injected to each mouse through the tail vein. As controls, MAs (5×10^6 cells, in 100 μL PBS) without NG loading, HA/DOX@PPy NGs (2.23 mg/mL, [DOX] = 42.9 μg/mL, in 100 μL PBS) with the same amount to the internalized NGs by MAs, and PBS (100 μL) were also respectively *i.v.* injected to the mice. After an 808-nm laser irradiation at the tumor region for 5 min (1.5 W/cm^2), thermal images and corresponding temperature variations of each mouse were recorded.

***In vivo* antitumor therapy.** *In vivo* treatments of tumors were initiated when the tumor size reached approximately 100 mm^3. The nude mice bearing 4T1 subcutaneous tumors were randomly divided into 5 groups with 5 mice in each group: PBS, free DOX, MAs-NGs, HA/DOX@PPy NGs + Laser and MAs-NGs +Laser. The day of the first injection treatment was considered to be day 0. Each mouse was intravenously injected with PBS (100 μL), free DOX (429 μg/mL, in 100 μL PBS), MAs-NGs (5×10^6 cells, [DOX] = 429 μg/mL, in 100 μL PBS), HA/DOX@PPy NGs + Laser (2.23 mg/mL, [DOX] = 429 μg/mL, in 100 μL PBS) and MAs-NGs + Laser on day 0, day 4 and day 8, respectively. In each treatment, 5×10^6 cells dispersed in 100 μL of PBS were intravenously injected to each mouse for MAs-NGs and MAs-NGs + Laser groups, and the injected DOX dosage in each group was kept consistent at 42.9 μg per mouse (1.95 mg/kg DOX for each mouse), which is significantly higher than the lowest typical injection dose (1 mg/kg) reported in the literature [4]. For PTT, the tumor of each mouse was irradiated by an 808-nm laser (1.5 W/cm^2, for 5 min) at 2 and 24 h post-injection, respectively for the HA/DOX@PPy NGs + Laser and MAs-NGs + Laser groups. The tumor volume

and body weight of each mouse were measured every other day for 15 days. At the 15th day, one mouse in each group was sacrificed and the tumor was removed from the sacrificed mouse for hematoxylin & eosin (H&E) and terminal deoxynucleotidyl transferase-mediated dUTP-biotin nick end labeling (TUNEL) staining. The remaining mice were used to record the survival rate. The tumor volume (V) was calculated according to the formula of $V = \text{tumor length} \times (\text{width})^2/2$.

***In vivo* biosafety examination.** First, to examine the histological changes of the mice, major organs including heart, liver, spleen, lung, and kidney from each mouse of different groups were harvested at 15th day post-treatment, fixed in 4% paraformaldehyde, embedded into paraffin and sectioned into thin slices before H&E staining. After that, the specimens were observed by inverted optical microscopy (Nikon Corporation, Tokyo, Japan).

To investigate whether MAs or MAs-NGs would induce systematic toxicity in normal nude mice ($n = 3$), the mice were euthanized on day 7 post-treatment with PBS, MAs (5×10^6 cells) or MAs-NGs (5×10^6 cells), and the whole blood were collected. Serum samples were prepared by centrifuging the blood samples at 4000 rpm for 15 min, which were stored at -80 °C and then subjected to biochemical analysis and the quantification of inflammation-related cytokines. The liver function (alanine transaminase (ALT), aspartate transaminase (AST)) and kidney function (urea nitrogen (BUN), serum creatinine (CREA)) indices were measured by a biochemical analyzer (Chemray 800, Rayto, Shenzhen, China). The inflammation cytokines (Interleukin-6 (IL-6), IL-12, interferon gamma (IFN-γ) and tumor necrosis factor-alpha (TNF-α)) were analyzed through ELLSA.

Statistical analysis. Experimental data were given as the mean ± standard deviation ($n \geq 3$). One-way analysis of variance method was adopted to evaluate the significance of the experimental data between groups. A p value of 0.05 was selected as the significance level, and all data were marked as (*) for $p < 0.05$, (**) for $p < 0.01$, and (***) for $p < 0.001$, respectively.

Table S1. Hydrodynamic size, zeta potential and polyderpersity index (PDI) of HA NGs prepared using sodium hyaluronate with different molecular wights *via* a double-emulsion method. Data are shown as mean ± SD (n = 3).

Molecule weight of HA (kD)	Hydrodynamic size (nm)	Zeta potential (mV)	PDI
48	243.8 ± 3.4	-7.9 ± 0.5	0.49 ± 0.48
320	316.7 ± 11.7	-4.5 ± 0.2	0.25 ± 0.06
950	383.0 ± 5.9	- 4.2 ± 0.3	0.22 ± 0.03

Table S2. Hydrodynamic size, zeta potential and polyderpersity index (PDI) of the HA@PPy NGs with different PPy loading degrees. Data are shown as mean ± SD (n = 3).

Samples	HA/Py mass ratio	Hydrodynamic size (nm)	Zeta potential (mV)	PDI
HA@PPy NGs Mw_{HA} = 48 kDa	1: 0.11	193.1 ± 3.0	-2.9 ± 0.3	0.11 ± 0.06
	1: 0.23	154.8 ± 8.1	4.6 ± 0.5	0.15 ± 0.04
	1: 0.46	128.8 ± 3.9	10.6 ± 0.7	0.12 ± 0.03
	1: 0.69	141.1 ± 1.9	7.8 ± 0.4	0.09 ± 0.06
	1: 0.92	276.5 ± 20.2	23.9 ± 0.4	1.00 ± 0.00
HA@PPy NGs Mw_{HA} = 320 kDa	1: 0.11	329.9 ± 18.9	1.8 ± 0.1	0.29 ± 0.04
	1: 0.23	248.5 ± 8.1	6.9 ± 0.5	0.20 ± 0.02
	1: 0.46	198.1 ± 4.9	9.7 ± 0.4	0.02 ± 0.02
	1: 0.69	292.9 ± 19.9	10.6 ± 0.6	0.15 ± 0.04
	1: 0.92	412.5 ± 14.3	13.9 ± 0.5	0.21 ± 0.02

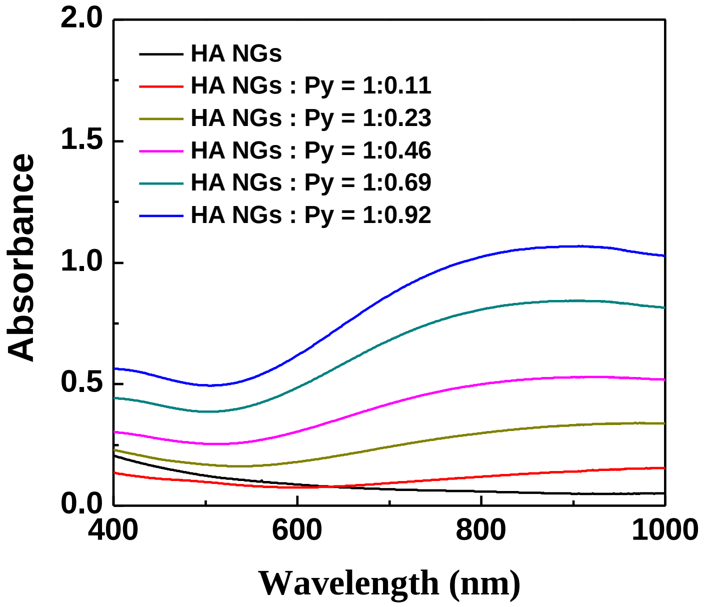

Figure S1. UV-vis spectra of HA NGs (Mw_{HA} = 320 kD) and HA@PPy NGs with different PPy loading degrees.

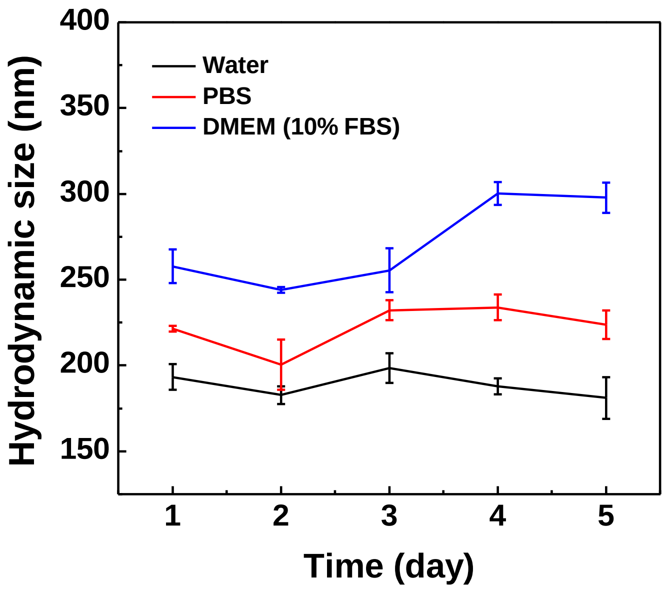

Figure S2. Hydrodynamic size change of HA@PPy NGs (Mw_{HA} = 320 kD, mass ratio of HA: Py = 1: 0.46) in water, PBS and DMEM containing 10% FBS over a period of 5 days (n = 3).

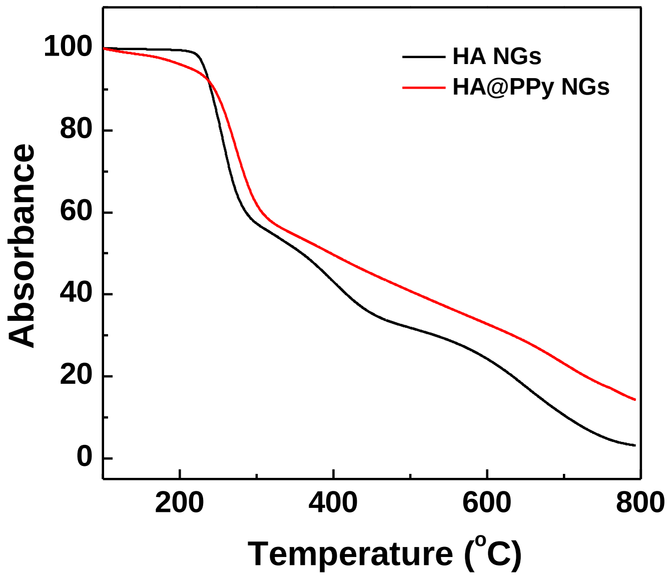

Figure S3. TGA analysis of HA NGs and HA@PPy NGs.

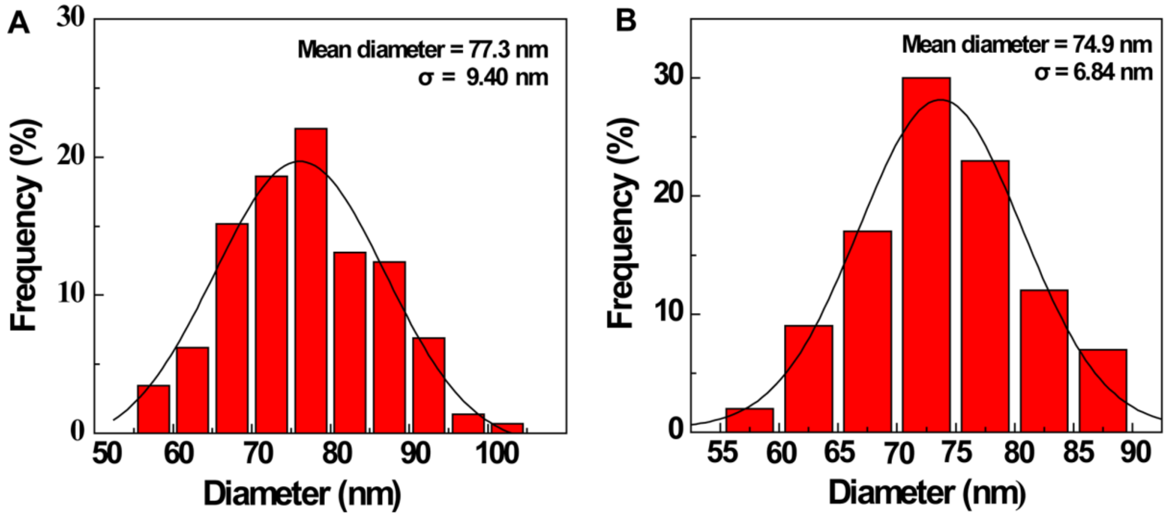

Figure S4. Size distribution histograms of HA@PPy NGs in SEM images (A) and TEM images (B), respectively.

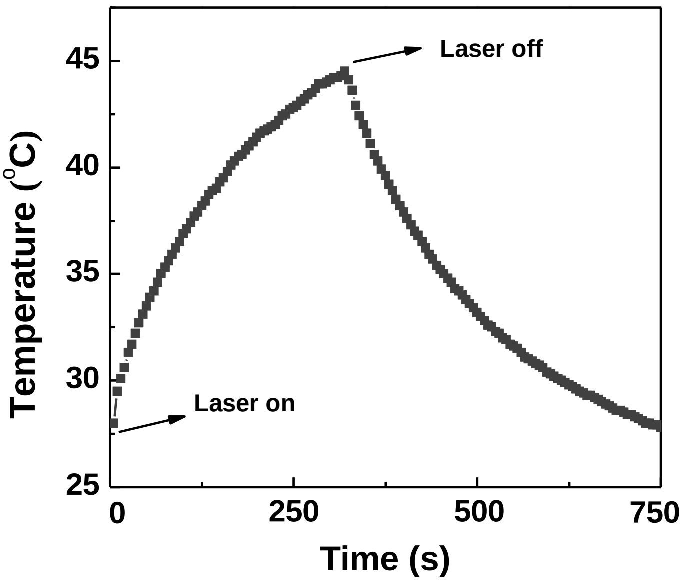

Figure S5. The heating-cooling curve of HA@PPy NGs (1 mg/mL) under an 808-nm laser irradiation at a power density of 1 W/cm^2.

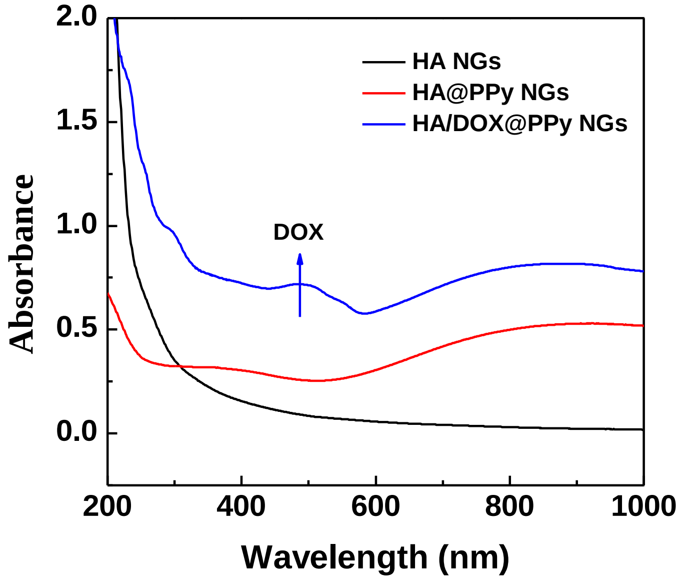

Figure S6. UV-vis spectra of HA NGs, HA@PPy NGs and HA/DOX@PPy NGs (Mw_{HA} = 320 kD, molar ratio of HA: Py = 1: 0.46).

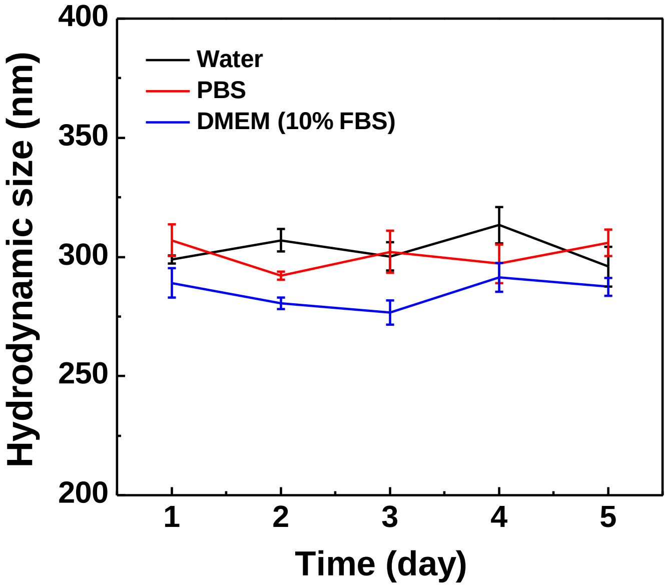

Figure S7. Hydrodynamic size change of HA/DOX@PPy NGs dispersed in water, PBS and DMEM containing 10% FBS over a period of 5 days (n = 3).

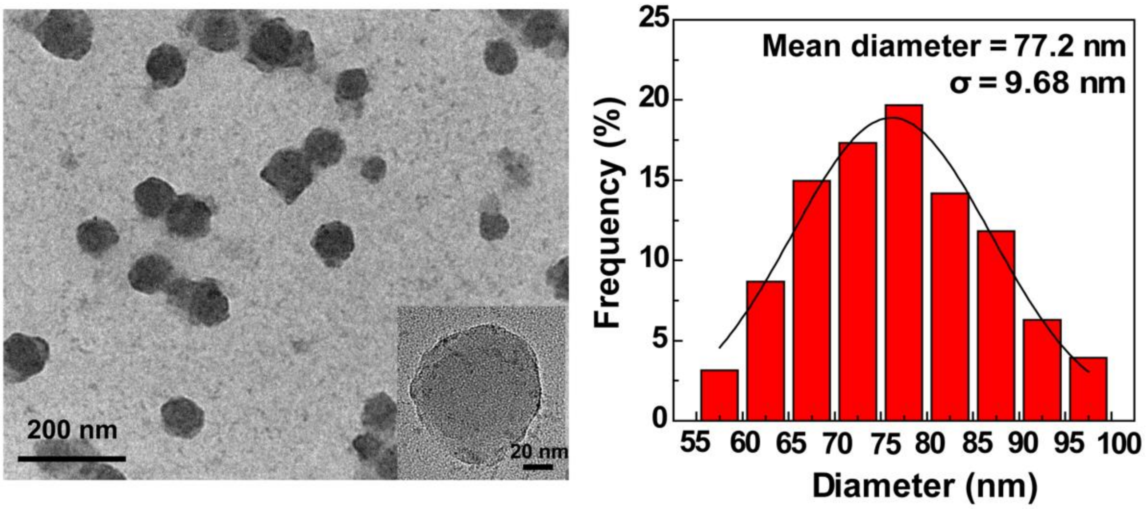

Figure S8. TEM image and size distribution of HA/DOX@PPy NGs.

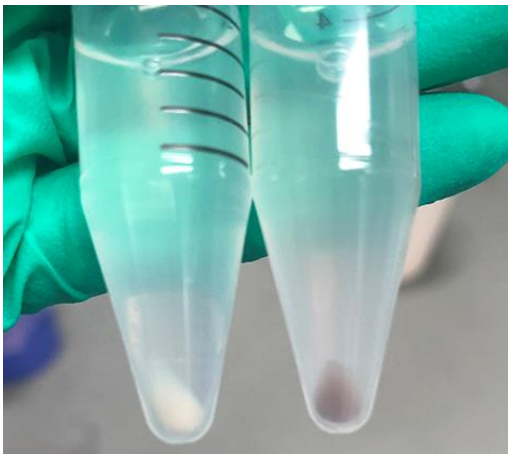

Figure S9. Photographs of MAs incubated without (Left) and with HA/DOX@PPy NGs (Right).

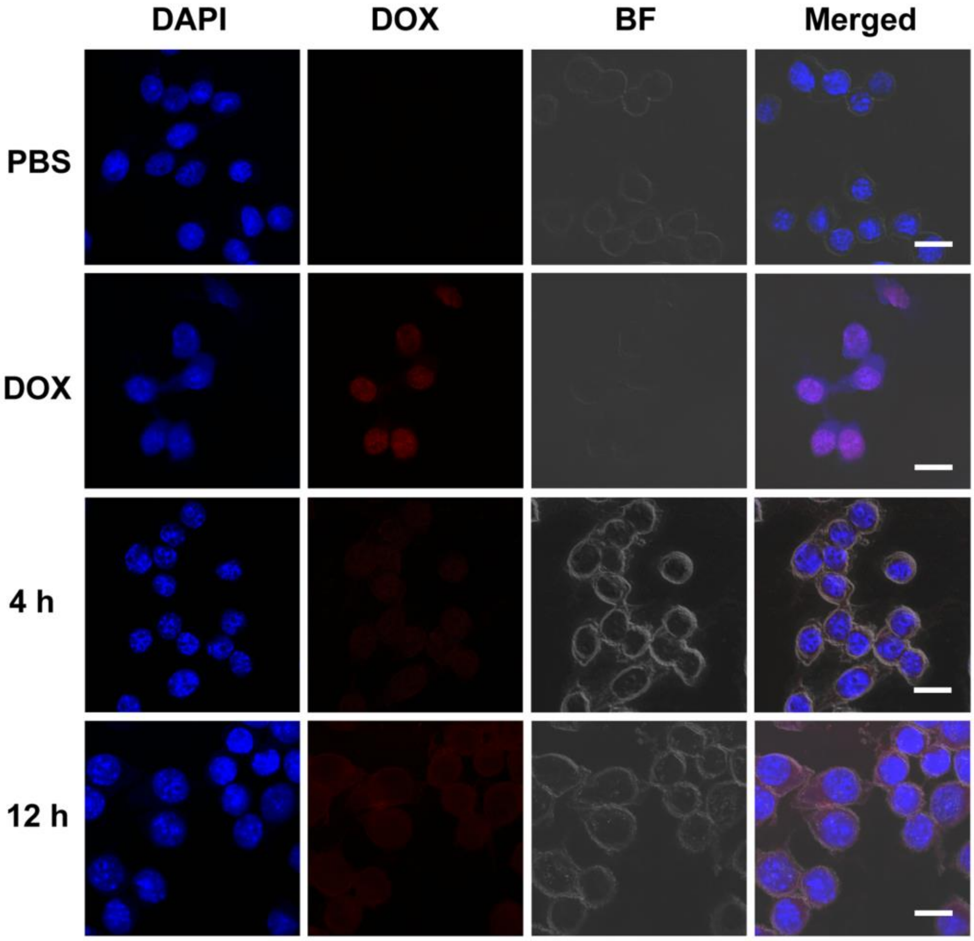

Figure S10. CLSM images of MAs treated with the HA/DOX@PPy NGs for 4 or 12 h. The MAs treated with PBS and free DOX for 4 h were used as controls. Scale bar in each panel represents 20 μm.

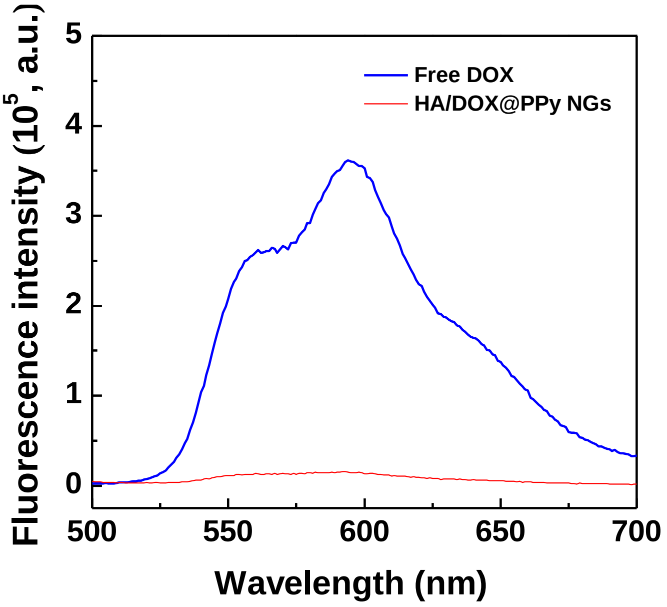

Figure S11. Fluorescence emission spectra of free DOX and HA/DOX@PPy NGs.

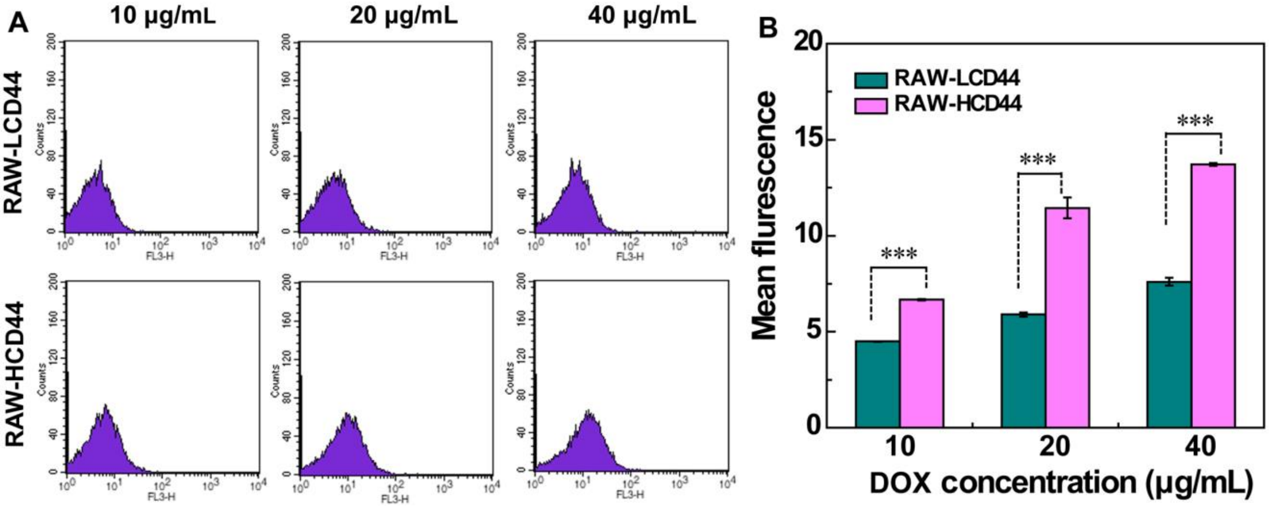

Figure S12. (A) FACS analysis of RAW-LCD44 (HA-blocked MAs) and RAW-HCD44 cells treated with the HA/DOX@PPy NGs at different DOX concentrations (10, 20, or 40 µg /mL) for 4 h. (B) Quantified mean fluorescence intensity of cells at different DOX concentrations.

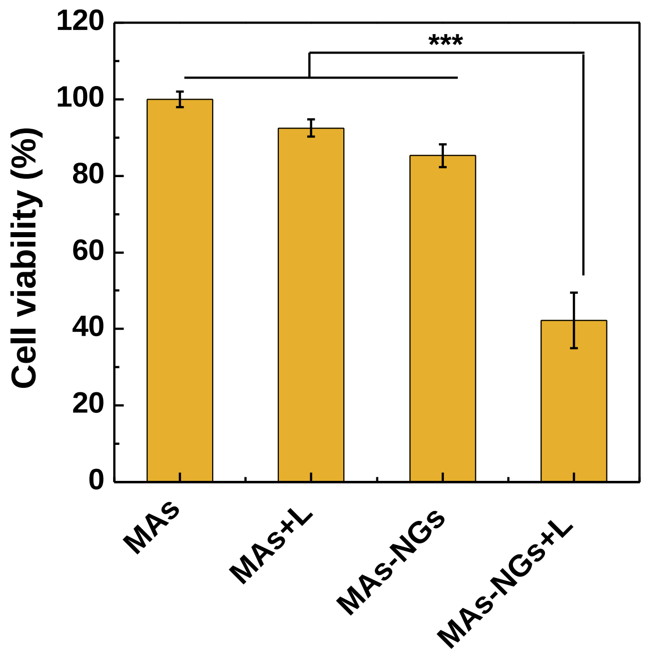

Figure S13. Viability of MAs and MAs-NGs at 24 h in the absence or presence (+ L) of NIR laser (808 nm) at a power density of 1.5 W/cm^2 for 5 min.

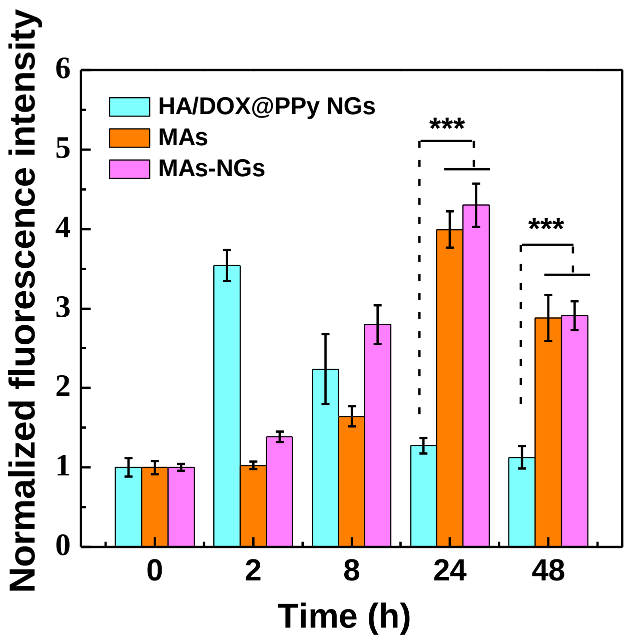

Figure S14. Semi-quantitative analysis of the fluorescence intensity of tumors at different time points post intravenous injection of the HA/DOX@PPy NGs, MAs and MAs-NGs.

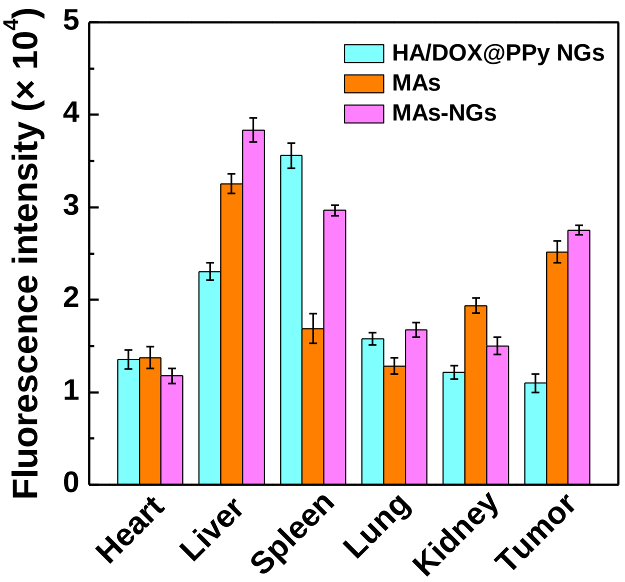

Figure S15. Mean fluorescence intensities of the *ex vivo* major organs and tumors at 48 h post-injection of HA/DOX@PPy, MAs or MAs-NGs.

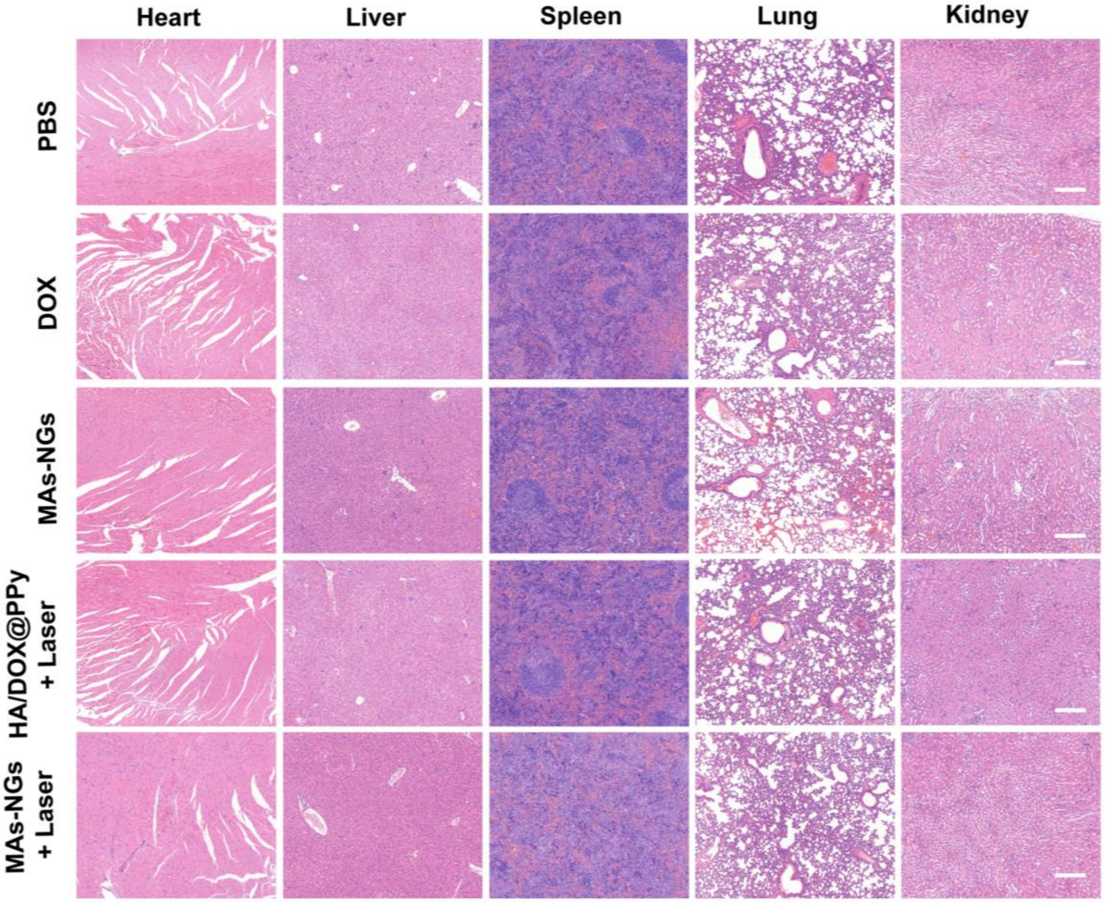

Figure S16. H&E staining of slices of the heart, liver, spleen, lung and kidney from 4T1 tumor-bearing nude mice after different treatments for 15 days. The scale bar in each panel represents 100 μm.

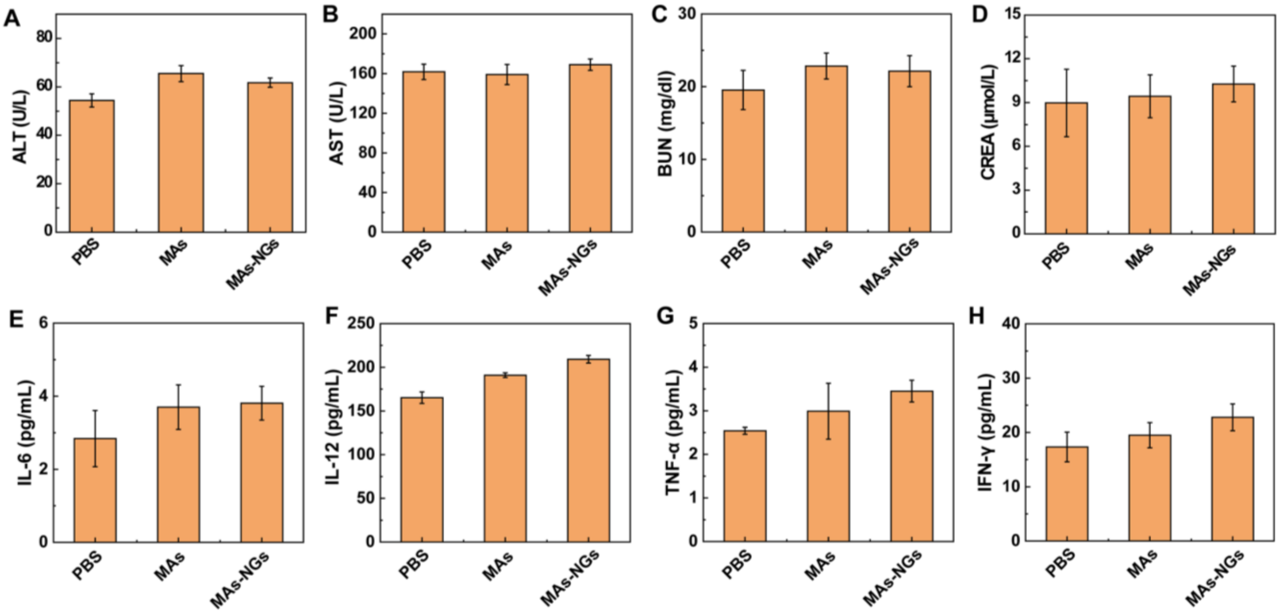

Figure S 17. Analysis of liver (A. ALT and B. AST) and kidney function indices (C. BUN and D. CREA) in serums that obtained from healthy nud mice at 7 day post-treatments. The concentrations of TNF-α, IFN-γ, IL-6 and IL-12 in serums that obtained from healthy nud mice at 7 day post-treatment with PBS, MAs or MAs-NGs and analyzed by ELISA. Data are presented as means ± SD.

References

1. Bhana S, Lin G, Wang LJ, Starring H, Mishra SR, Liu G, et al. Near-Infrared-absorbing gold nanopopcorns with iron oxide cluster core for magnetically amplified photothermal and photodynamic cancer therapy. ACS Appl Mater Interfaces. 2015; 7: 11637-47.

2. Lu SY, Li X, Zhang JL, Peng C, Shen MW, Shi XY. Dendrimer-stabilized gold nanoflowers embedded with ultrasmall iron oxide nanoparticles for multimode imaging-guided combination therapy of tumors. Adv Sci. 2018; 5: 1801612.

3. Huang H, Jiang CT, Shen S, Liu A, Gang YJ, Tong QS, et al. Nanoenabled reversal of IDO1-mediated immunosuppression synergizes with immunogenic chemotherapy for improved cancer

therapy. Nano Lett. 2019; 19: 5356-65.

4. Zhang WZ, Wang MZ, Tang W, Wen R, Zhou SY, Lee CB, et al. Nanoparticle-laden macrophages for tumor-tropic drug delivery. Adv Mater. 2018; 30: 1805557.
